# Intrinsic bias at non-canonical, β-arrestin-coupled seven transmembrane receptors

**DOI:** 10.1016/j.molcel.2021.09.007

**Published:** 2021-09-27

**Authors:** Shubhi Pandey, Punita Kumari, Mithu Baidya, Ryoji Kise, Yubo Cao, Hemlata Dwivedi-Agnihotri, Ramanuj Banerjee, Xaria X. Li, Cedric S. Cui, John D. Lee, Kouki Kawakami, Jagannath Maharana, Ashutosh Ranjan, Madhu Chaturvedi, Gagan Deep Jhingan, Stéphane A. Laporte, Trent M. Woodruff, Asuka Inoue, Arun K. Shukla

**Affiliations:** 1 Department of Biological Sciences and Bioengineering, Indian Institute of Technology, Kanpur 208016, India; 2 Graduate School of Pharmaceutical Sciences, Tohoku University, Sendai, Miyagi 980-8578, Japan; 3 Department of Pharmacology and Therapeutics, McGill University, Montréal, QC H3G 1Y6, Canada; 4 School of Biomedical Sciences, Faculty of Medicine, The University of Queensland, Brisbane 4072, Australia; 5 Valerian Chem Pvt. Ltd., Vproteomics, New Delhi 110049, India; 6 Department of Medicine, McGill University Health Center, McGill University, Montréal, QC H4A 3J1, Canada; 7 Queensland Brain Institute, The University of Queensland, Brisbane 4072, Australia

## Abstract

G-protein-coupled receptors (GPCRs), also known as seven transmembrane receptors (7TMRs), typically interact with two distinct signal-transducers, i.e., G proteins and β-arrestins (βarrs). Interestingly, there are some non-canonical 7TMRs that lack G protein coupling but interact with barrs, although an understanding of their transducer coupling preference, downstream signaling, and structural mechanism remains elusive. Here, we characterize two such non-canonical 7TMRs, namely, the decoy D6 receptor (D6R) and the complement C5a receptor subtype 2 (C5aR2), in parallel with their canonical GPCR counterparts. We discover that D6R and C5aR2 efficiently couple to βarrs, exhibit distinct engagement of GPCR kinases (GRKs), and activate non-canonical downstream signaling pathways. We also observe that βarrs adopt distinct conformations for D6R and C5aR2, compared to their canonical GPCR counterparts, in response to common natural agonists. Our study establishes D6R and C5aR2 as βarr-coupled 7TMRs and provides key insights into their regulation and signaling with direct implication for biased agonism.

## Graphical abstract

**Graphical abstract:**
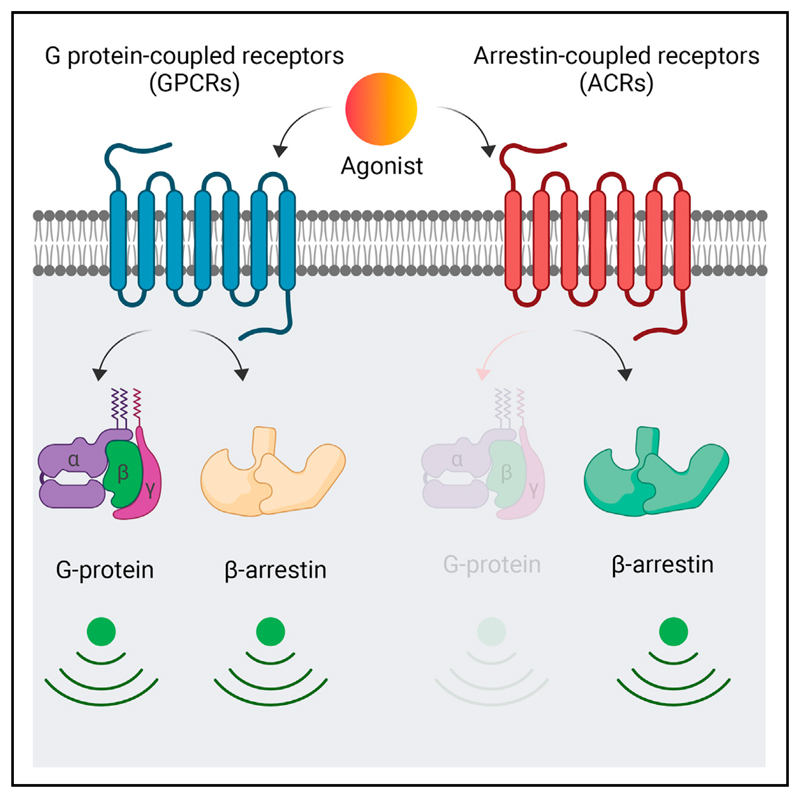

## Introduction

G-protein-coupled receptors (GPCRs), also referred to as seven transmembrane receptors (7TMRs), constitute a large family of cell surface proteins in the human genome with direct involvement in all major physiological processes (Pierce et al., 2002; Rosenbaum et al., 2009). The overall transducer coupling framework of these receptors is highly conserved across the family in which agonist activation results in the coupling of heterotrimeric G proteins followed by phosphorylation, primarily by GPCR kinases (GRKs), at multiple sites, and subsequent binding of β-arrestin 1 and 2 (βarr1 and 2; also known as arrestin2 and 3) (Reiter et al., 2012; Weis and Kobilka, 2018). Although natural agonists typically induce both G protein and βarr coupling to these receptors, it is possible to design ligands that promote preferential coupling to one of these transducers leading to biased signaling (Rajagopal et al., 2010b; Smith et al., 2018). This framework, referred to as biased agonism, is considered to harbor previously untapped therapeutic potential for minimizing the side effects exerted by conventional GPCR-targeting drugs (Ranjan et al., 2017; Violin and Lefkowitz, 2007; Whalen et al., 2011).

A central question that still remains to be answered unequivocally is whether naturally biased 7TMRs, which are able to engage one of the two well-known transducers selectively and exclusively, i.e., G proteins and βarrs, exist. Although there are scattered examples in the literature of 7TMRs, which lack functional G protein coupling but exhibit agonist-induced βarr recruitment (Bachelerie et al., 2014; Nibbs and Graham, 2013; Van Lith et al., 2009), they are poorly characterized in terms of a comprehensive G-protein-coupling profile, GRK dependence, βarr conformational signatures, and downstream signaling. These receptors include, for example, the human decoy D6 receptor (D6R) (Bonecchi et al., 2004; Borroni et al., 2013; Weber et al., 2004), the chemokine receptor CXCR7 (Rajagopal et al., 2010a), and the complement C5a receptor (C5L2/C5aR2) (Kalant et al., 2005; Li et al., 2019; Pandey et al., 2019a, 2020). Interestingly, such receptors share a natural agonist with prototypical GPCRs and, therefore, constitute an intriguing pair of receptors activated by a common agonist that exhibit strikingly different transducer coupling patterns. For example, the complement C5a peptide binds to two different 7TMRs, namely, C5aR1 and C5aR2, but only C5aR1 exhibits functional coupling to G proteins, whereas both of them recruit βarrs (Li et al., 2020a; Okinaga et al., 2003).

**Figure 1:**
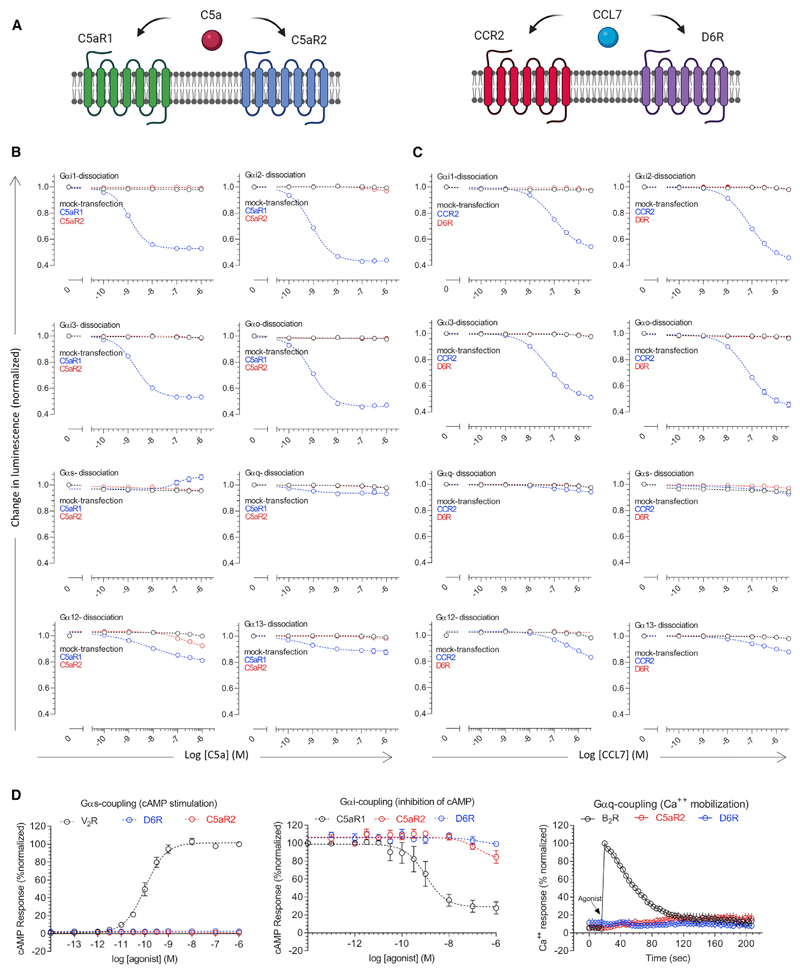
D6R and C5aR2 lack functional G protein coupling (A) Schematic representation of canonical GPCR and non-canonical 7TMR pairs activated by a common agonist. C5a, complement C5a; C5aR1, C5a receptor subtype 1; C5aR2, C5a receptor subtype 2; CCL7, chemokine CCL7; CCR2, C-C chemokine receptor subtype 2; D6R, decoy D6 receptor. (B and C) Agonist-induced dissociation of heterotrimeric G proteins for C5aR1-C5aR2 and CCR2-D6R pairs measured using NanoBiT complementation assay. Data (mean ± SEM) represent three independent experiments, normalized with respect to baseline signal (i.e., vehicle treatment). (D) Agonist-induced second messenger response measured using the GloSensor assay (cAMP stimulation and inhibition of forskolin-induced cAMP level) and Fluo-4 NW calcium mobilization assay. For each of the second messenger assays, a well-established prototypical GPCR was included as a reference (V_2_R, vasopressin receptor subtype 2; B_2_R, bradykinin receptor subtype 2). Data (mean ± SEM) represent four independent experiments, normalized with respect to maximum signal (treated as 100%). See also Figure S1.

Here, we set out to characterize two such receptor pairs, namely, the CCR2-D6R activated by a common chemokine ligand, CCL7, and C5aR1-C5aR2 that share complement C5a as their native agonist (Figure 1A). We discover that D6R and C5aR2 do not couple to any of the common G proteins but robustly recruit βarrs, they have differential dependence on GRKs, activate a broad spectrum of potential signaling pathways, and impart distinct conformational signatures on βarrs compared to their prototypical GPCR counterparts. This study not only establishes D6R and C5aR2 as βarr-coupled 7TM receptors but also provides a conceptual and experimental framework that can be leveraged to discover additional examples of naturally biased receptors, and to better understand the intricacies of biased agonism and 7TMR signaling.

## Results

### Lack of functional G protein coupling to D6R and C5aR2

Previous studies have suggested that D6R and C5aR2 lack G protein coupling; however, the experimental evidence is limited primarily to lack of cyclic AMP (cAMP) response as a readout of Gαi-activation (Borroni et al., 2013; Okinaga et al., 2003). Therefore, we first measured G protein activation profile of these receptors using a NanoBiT-based G protein dissociation assay (Inoue et al., 2019). Here, a NanoBiT-G protein consisting of a large fragment (LgBiT)-containing Gα subunit and a small fragment (SmBiT)-fused Gγ2 subunit, along with the untagged Gβ1 subunit are expressed in HEK293 cells together with the receptor (Inoue et al., 2019). Subsequently, the agonist-induced decrease in luminescence resulting from the dissociation of Gα and Gβγ sub-units is measured as a readout of G protein activation (Inoue et al., 2019). We used CCR2 and C5aR1 as prototypical counterparts of D6R and C5aR2, respectively, and the receptors from each pair were expressed at comparable levels (Figure S1A). We observed that D6R and C5aR2 do not elicit a robust response for any of the major G protein subtypes tested here, although there is a slight dissociation of Gα12 for C5aR2 at higher C5a concentrations (Figures 1B and 1C), whereas CCR2 and C5aR1 yielded efficient activation of the Gαi subtype. Moreover, D6R and C5aR2 also failed to elicit any detectable second messenger response in cAMP and Ca^2+^ mobilization assays (Figure 1D). Taken together, these data demonstrate the lack of measurable activation of common G proteins upon agonist stimulation of D6R and C5aR2.

### βarr recruitment, trafficking, and GRK preference for D6R and C5aR2

**Figure 2:**
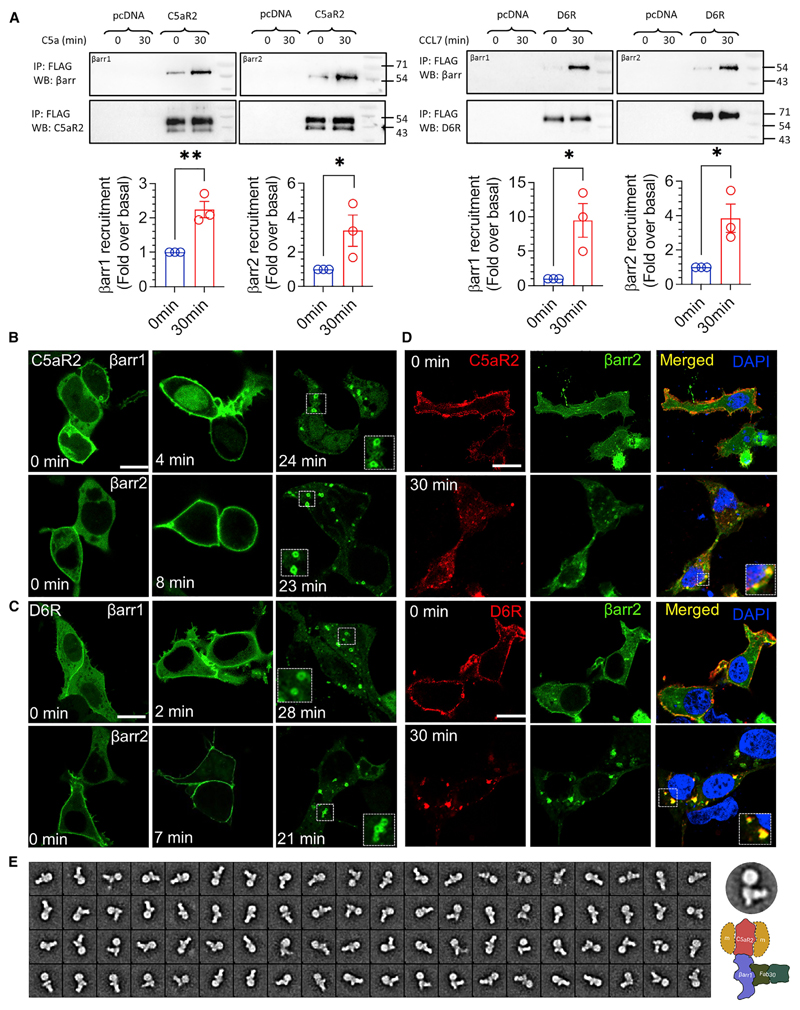
D6R and C5aR2 recruit βarrs upon agonist stimulation (A) A co-immunoprecipitation experiment using lysate prepared from HEK293 cells expressing indicated receptors and purified βarr1/2 followed by western blotting reveal the interaction of D6R and C5aR2 with βarrs. A representative blot and densitometry-based quantification from three independent experiments analyzed using unpaired t test (*p < 0.05; **p < 0.01) are presented. Two bands in C5aR2 blots correspond to mature (glycosylated) and immature (non-glycosylated) receptor populations. (B and C) Agonist-induced (CCL7, 100 nM; C5a, 100 nM) trafficking of βarrs was monitored in HEK293 cells expressing the indicated receptor and mYFP-tagged βarrs using confocal microscopy at the indicated time points. Representative images from three independent experiments are shown (scale bar, 10 μm). (D) Internalized D6R and C5aR2 (stained using DyLight-594 conjugated anti-FLAG M1 antibody) co-localize with βarr2-mYFP as monitored by confocal microscopy on fixed and permeabilized HEK293 cells. Representative images from three independent experiments are shown (scale bar,10 μm), and the Pearson’s correlation coefficient (PCC) was 0.68 ± 0.05,0.72 ± 0.06, 0.94 ± 0.01, and 0.96 ± 0.01 for C5aR2, 0 min (12 cells); C5aR2,10 min (13 cells); D6R, 0 min (14 cells); and D6R, 10 min (15 cells), respectively. (E) Single-particle analysis of the C5aR2-βarr1-Fab30 complex isolated from *Sf9* cells expressing C5aR2, GRK6, and βarr1 further corroborates the interaction of βarr1 with C5aR2. The complex was isolated using M1 antibody affinity purification and size-exclusion chromatography, followed by negative-staining-based single-particle analysis. 2D class averages from approximately 10,000 particles are shown here, and a typical 2D class average is indicated together with a schematic representation of the complex. See also Figures S1 and S2.

In order to assess βarr recruitment to D6R and C5aR2, we first used a co-immunoprecipitation (coIP) assay by expressing these receptors in HEK293 cells followed by agonist stimulation, addition of purified βarrs, and chemical cross-linking. We observed a robust interaction between βarr1 and 2 upon agonist stimulation with each of these receptors (Figure 2A; Figures S1B–S1F). Furthermore, co-expression of βarrs together with receptors followed by chemical cross-linking and coIP also corroborated the agonist-induced βarr interaction with D6R and C5aR2 (Figures S2A and S2B). Next, we monitored agonist-induced trafficking of mYFP-tagged βarrs for D6R and C5aR2 by using confocal microscopy. We observed that upon agonist stimulation, βarrs were first localized to the plasma membrane followed by their trafficking to endosomal vesicles (Figures 2B and 2C). Interestingly, we also observed some level of βarr localization to the membrane in D6R- and C5aR2-expressing cells even under basal conditions (i.e., before agonist stimulation), which is more pronounced for βarr2 (Figures 2B and 2C). We further corroborated agonist-induced βarr1 and 2 trafficking and pre-coupling by using mCherry-tagged βarr constructs in confocal microscopy and scoring βarr1/2 localization patterns from a pool of cells (Figures S2C–S2F).

To probe whether internalized vesicles harbor both βarrs and receptors, we measured their colocalization by immunostaining and observed that both D6R and C5aR2 co-localized on endosomal vesicles together with βarr2 (Figure 2D). These findings suggest that the agonist-induced βarr interaction of D6R and C5aR2 has a functional consequence in terms of driving their endocytosis. In order to further establish the βarr interaction, we also reconstituted the C5aR2-βarr1 complex stabilized by a synthetic antibody fragment (Fab30) directed against βarr1 and subjected the complex to single-particle negative-staining-based visualization by electron microscopy. As presented in Figure 2E, we observed several 2D class averages reminiscent of a previously described tail-engaged receptor-βarr interaction (Shukla et al., 2014), which further confirms a direct interaction between C5aR2 and βarr1.

**Figure 3:**
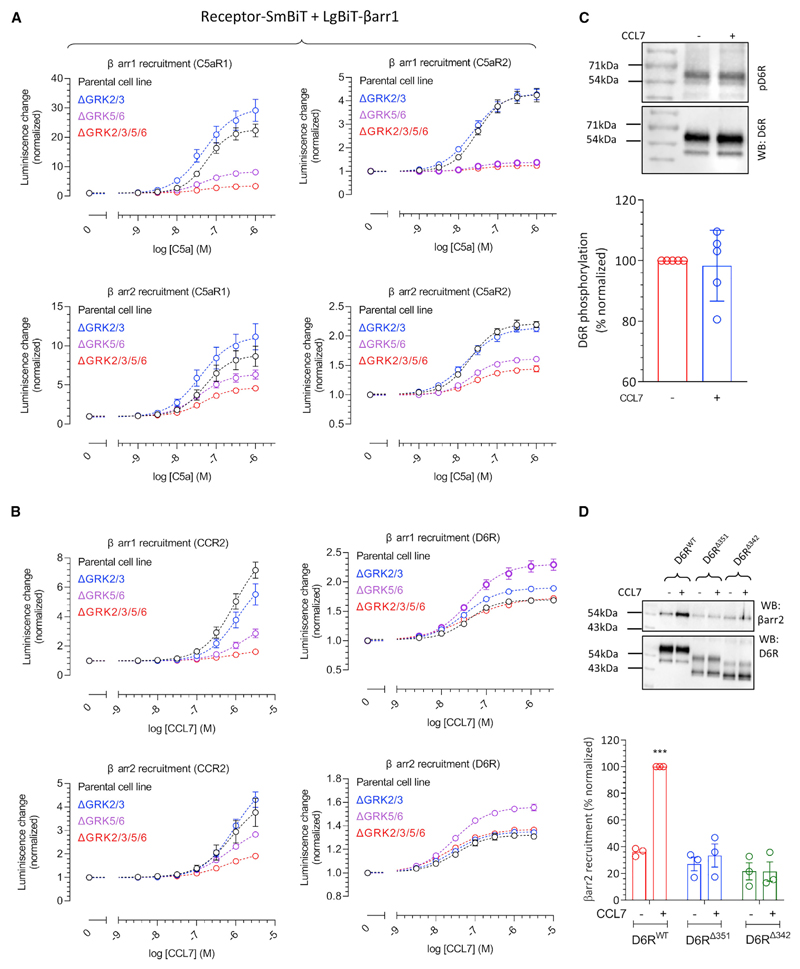
D6R and C5aR2 exhibit a distinct GRK preference for βarr recruitment (A and B) HEK293 cells lacking indicated GRKs were transfected with the indicated receptor and βarrs followed by measurement of agonist-induced βarr recruitment by using the NanoBiT assay. Data (mean ± SEM) from three independent experiments are normalized with respect to basal signal (i.e., vehicle treatment). (C) HEK293 cells expressing D6R were stimulated with CCL7 (100 nM), and total receptor phosphorylation was assessed by using the pIMAGO phospho-protein detection kit. A representative blot and densitometry-based quantification (mean ± SEM) from five independent experiments normalized with respect to basal signal are presented. (D) Carboxyl-terminal truncation reduces the βarr2 interaction with D6R as assessed by a co-immunoprecipitation experiment using HEK293 cells expressing the indicated receptor construct and βarr2 followed by western blotting. A representative blot and densitometry-based quantification (mean ± SEM), normalized with D6R^WT^ stimulation condition (treated as 100%), is presented (n = 3; one-way ANOVA; p < 0.001). See also Figures S3 and S4.

As receptor phosphorylation is a key determinant for βarr recruitment and GRKs play a central role in this process, we measured the contribution of different GRKs in agonist-induced βarr recruitment by using CRISPR-Cas9-based GRK knockout cells (Arveseth et al., 2021; Figure S3C). These assays were first performed under Gαi/Gαo-inhibited conditions by co-expressing the catalytic subunit of pertussis toxin (PTX) in order to compare the responses for each of the receptors in the absence of G protein signaling. We observed that C5aR2 primarily relies on GRK5/6 for βarr recruitment, a pattern that is mostly analogous to C5aR1 (Figure 3A). On the contrary, we observed that GRK knockout does not have a substantial effect on CCL7-induced βarr recruitment for D6R and even an increase in βarr recruitment upon GRK5/6 knockout (Figure 3B). This finding is in striking contrast with CCR2 results, which show that CCR2 clearly requires GRK5/6 for βarr recruitment (Figure 3B). We also note here that agonist-induced βarr recruitment for D6R is significantly lower than that for CCR2 in terms of fold increase over basal response in this assay, although the potency of CCL7 for D6R is higher than that for CCR2 (Figure 3B). This relatively smaller agonist-induced response may reflect the basal recruitment of βarrs to D6R as also observed in confocal microscopy (Figure 2C).

As GRK2/3 are known to be targeted to the membrane and activated by the free Gβγ subunit (Pitcher et al., 1992), we also measured βarr1/2 recruitment in the parent and ΔGRK5/6 cells in the presence and absence of PTX (Figures S3A and S3B). Interestingly, we observed that PTX had a significant inhibitory effect on βarr recruitment to C5aR1 in ΔGRK5/6 cells but not in parent cells (Figure S3A). In contrast, there was no measurable effect of PTX on βarr recruitment to C5aR2 in either of the cell lines (Figure S3A). On the other hand, PTX treatment had a measurable effect on CCR2-βarr recruitment in both cell lines, although the effect was more pronounced in the ΔGRK5/6 cell line (Figure S3B). Similar to C5aR2, there was no substantial effect of PTX on CCL7-induced βarr recruitment to D6R under these conditions (Figure S3B). Taken together, these data suggest that C5aR1 and CCR2 use both GRK2/3 and GRK5/6 for βarr recruitment, whereas C5aR2 depends primarily on GRK5/6. This result also agrees with the lack of G protein coupling for C5aR2, which potentially limits the Gβγ-mediated membrane recruitment of GRK2/3.

In order to probe the interesting observation that the depletion of GRKs does not reduce the D6R-βarr interaction, we assessed agonist-induced phosphorylation of D6R. We observed that D6R exhibits robust constitutive phosphorylation, which does not change significantly upon CCL7 stimulation (Figure 3C). As a control, we also measured the phosphorylation of a chimeric β2-adrenergic receptor with V2R carboxyl-terminus (referred to as β2V2R), and we observed an agonist-induced increase in phosphorylation as anticipated (Figure S4A). D6R harbors a number of Ser/Thr residues in its carboxyl terminus that represent potential phosphorylation sites (Figure S4B). In addition, it also harbors a stretch of acidic amino acids at its distal carboxyl terminus that has been suggested to play a role in its constitutive internalization (Galliera et al., 2004). Therefore, we generated two different truncations of D6R lacking either the distal region with acidic residues (D6R^Δ351^) or the Ser/Thr cluster and the acidic residue containing stretch together (D6R^Δ342^) (Figure S4B). D6R typically exhibits two bands on western blots in which the prominent upper band likely represents the glycosylated/mature receptor population, whereas the lower band of weaker intensity represents the partially glycosylated/immature receptor population. Interestingly, we observed that C terminus truncation of D6R resulted in a larger proportion of the lower band than D6R^WT^ (Figure S4D), which was also reflected in their lower surface expression. Therefore, we first normalized the surface expression of all three constructs (i.e., D6R^WT^, D6R^Δ351^, and D6R^Δ342^) to comparable levels (Figure S4C) by titrating the amount of transfected DNA followed by measuring their phosphorylation and βarr recruitment. We observed that D6R^Δ351^ is also constitutively phosphorylated similarly to D6R^WT^; however, D6R^Δ342^ did not exhibit constitutive phosphorylation (Figures S4D and S4E). These data indicate that receptor phosphorylation is localized primarily in the Ser/Thr cluster region i.e., between Ser^342^ and Ser^351^. We then measured the agonist-induced βarr2 interaction and trafficking for these truncated constructs,and observed that even D6R^Δ351^ exhibits a near-complete loss of βarr2 recruitment and trafficking, similar to D6R^Δ342^ (Figure 3D). These data indicate that D6R recruits βarrs primarily through the distal stretch in its carboxyl terminus containing acidic residues, despite having constitutive phosphorylation. In line with this observation, we also found that CCL7 stimulation fails to elicit any measurable trafficking of βarr2 for D6R^Δ351^ and D6R^Δ342^ mutants (Figures S4F and S4G). Taken together, these data help reconcile the intriguing observation that GRK knockout does not influence the βarr interaction for D6R.

### Distinct conformational signatures of barrs for D6R and C5aR2

**Figure 4:**
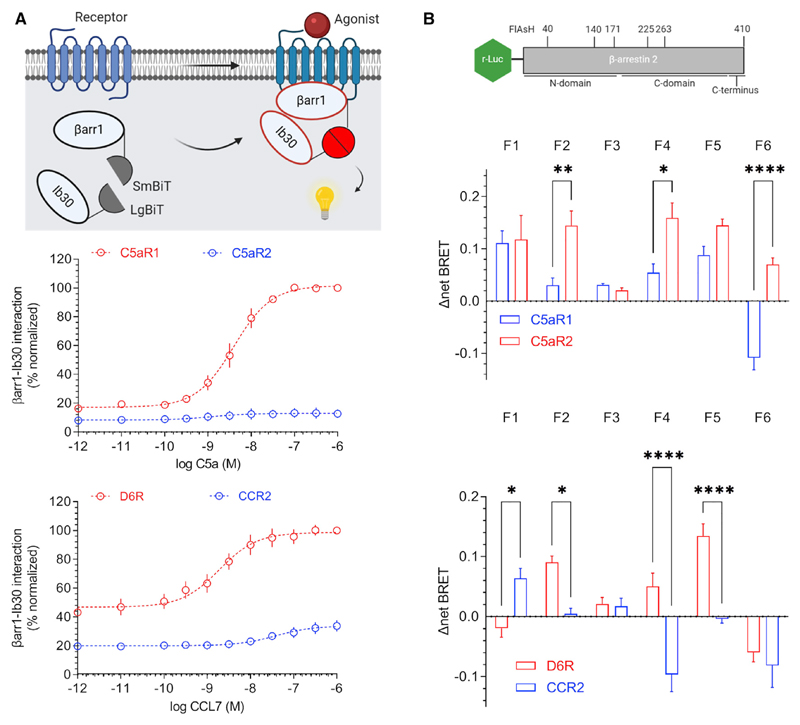
D6R and C5aR2 impart distinct conformations in βarrs (A) Intrabody30-based conformational sensor developed in the NanoBiT format (top panel) reveals distinct conformations of βarr1 for C5aR1-C5aR2 and D6R-CCR2 pairs. HEK293 cells expressing the indicated receptor, LgBiT-Ib30, and SmBiT-βarr1 were stimulated with various concentrations of agonists, and the luminescence signal was monitored. Data (mean ± SEM) from four independent experiments, normalized with respect to the maximal response (at 1 μM agonist concentration) in the receptor pairs, i.e., C5aR1 in the C5aR1-C5aR2 pair and D6R in the D6R-CCR2 pair (treated as 100%), are presented. (B) Intramolecular BRET sensors of βarr2 reveal distinct conformational signatures in βarr2 for C5aR1 versus C5aR2 and D6R versus CCR2. The top panel shows the schematic of sensors where the N terminus of βarr2 harbors r-Luc (Renilla luciferase) as the BRET donor, whereas the FlAsH motif (as BRET acceptor) is encoded in various positions in βarr2. HEK293 cells expressing the indicated receptor and sensor constructs were labeled with the FlAsH reagent followed by agonist stimulation and measurement of the BRET signal. Data (mean ± SEM) from four independent experiments are presented (two-way ANOVA; *p < 0.05, **p < 0.01, ****p < 0.0001).

In order to probe if the distinct transducer-coupling preference of D6R and C5aR2 with respect to their prototypical GPCR counterparts may impart distinct βarr conformations, we measured the conformational signatures of βarrs upon their interaction with these receptors. First, we used a previously described intrabody30 (Ib30)-based sensor for βarr1, which selectively recognizes receptor-bound conformation of βarr1 and reports agonist-induced formation of the receptor-βarr1 complex in cellular context (Baidya et al., 2020a; Baidya et al., 2020b). We observed that the Ib30 sensor reacted robustly to βarr1 upon C5a stimulation of C5aR1, but it failed to exhibit a strong response for C5aR2 under normalized surface expression of the receptors (Figure 4A). As C5aR2 robustly recruits βarr1, the lack of Ib30 sensor reactivity indicates a distinct conformation in C5aR2-bound βarr1 compared to C5aR1-bound βarr1. On the other hand, Ib30 recognized βarr1 for both D6R and CCR2, although the response was significantly weaker for CCR2 (Figure 4A). Considering the relatively stronger βarr1 recruitment to CCR2 than to D6R in a NanoBiT assay (Figure 3B), it is plausible that the difference in Ib30 sensor reactivity reflects distinct conformations of βarr1 for D6R and CCR2; however, further studies are required to probe this possibility. Collectively, the Ib30 sensor data also suggest that βarr1 conformations differ between the C5aR2 and D6R, which underscores the conformational diversity that exists in 7TMR-βarr complexes.

Next, we used FlAsH-BRET-based sensors of βarr2 (Lee et al., 2016) to probe the conformations of βarr2 in complex with these receptors. These intramolecular sensors harbor a BRET donor (Renilla-luciferase) at the N terminus of βarr2, whereas FlAsH label sequences (tetracysteine motifs) at different positions (Figure 4B). Thus, an in-parallel comparison of these sensors for a given receptor can reveal conformational signatures of βarr2 with a change in BRET signal as the readout. As presented in Figure 4B, we observed striking differences not only in C5aR1-C5aR2 and D6R-CCR2 pairs but also between C5aR2 and D6R. For example, there is an opposite change in BRET signal for the F6 sensor upon activation of C5aR1 versus C5aR2 (Figure 4B), whereas the F4 sensor displays a directionally opposite change in BRET signal for D6R versus CCR2 (Figure 4B). Furthermore, the comparison of BRET response for F1 and F6 sensors also reveals a distinct pattern for C5aR2 versus D6R (Figure 4B). Taken together, these data further corroborate the conformational differences in βarr1 revealed by the Ib30 sensor and collectively establish distinct βarr conformations induced by D6R and C5aR2 compared to their canonical GPCR counterparts. It is worth noting that these assays are rather qualitative in nature for assessing differences in receptor-βarr conformations, as they do not directly illuminate the precise differences in βarr conformations, and therefore, future studies using direct biophysical approaches are required.

### D6R and C5aR2 display distinct profiles of ERK1/2 phosphorylation

**Figure 5:**
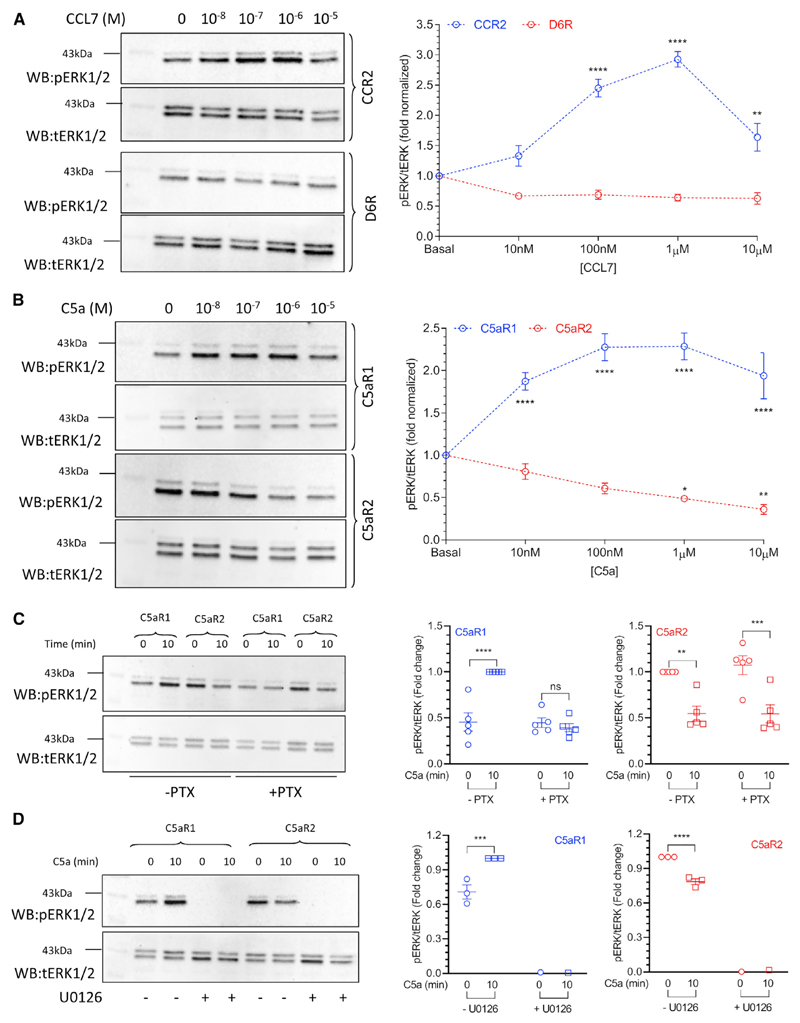
D6R and C5aR2 exhibit distinct patterns of ERK1/2 phosphorylation (A) CCL7 stimulation leads to robust ERK1/2 phosphorylation in HEK293 cells expressing CCR2; however, it fails to elicit any detectable ERK1/2 phosphorylation for D6R, as measured by western blotting. (B) C5aR1 stimulation exhibits a typical ERK1/2 phosphorylation pattern upon agonist stimulation, whereas C5aR2 displays an elevated level of basal ERK1/2 phosphorylation, which decreases upon C5a stimulation. (C) PTX treatment inhibits C5a-induced ERK1/2 phosphorylation downstream of C5aR1, but it fails to inhibit the elevated level of basal ERK1/2 phosphorylation for C5aR2. (D) Treatment with U0126, a MEK inhibitor, completely abolishes ERK1/2 phosphorylation for both C5aR1 and C5aR2, suggesting the involvement of a canonical mechanism of ERK1/2 phosphorylation. The left panels show representative blots, and the right panels present densitometry-based quantification (mean ± SEM) from 3–5 independent experiments analyzed using two-way ANOVA (*p < 0.05, **p < 0.01, ***p < 0.001, ****p < 0.0001). See also Figures S3 and S4.

Agonist-induced ERK1/2 phosphorylation has been one of the most common readouts of βarr signaling, and therefore, we assessed whether D6R and C5aR2 may stimulate ERK1/2 phosphorylation. Although CCL7 stimulation resulted in a robust increase in ERK1/2 phosphorylation downstream of CCR2, we did not observe a detectable stimulation for D6R-expressing cells (Figure 5A). We also observed a decrease in CCR2-mediated pERK1/2 at a high dose of CCL7 that has been reported previously for some chemokine receptors. Interestingly, we observed a typical pattern of ERK1/2 phosphorylation upon stimulation of C5aR1; however, we noticed an elevated level of pERK1/2 in C5aR2-expressing cells, which was reduced significantly upon C5a stimualtion in a dose-dependent fashion (Figure 5B). Interestingly, the elevated level of phospho-ERK1/2 was not sensitive to pre-treatment with PTX (i.e., Gαi inhibition) (Figure 5C) but it was ablated completely with U0126 (MEK inhibitor) pre-treatment (Figure 5D). These data suggest that although a canonical pathway involving MEK is involved in the enhanced level of phospho-ERK1/2, it is not dependent on Gαi. As we observed a measurable level of constitutive βarr localization in the membrane for C5aR2-expressing cells, we measured the effect of βarr knockdown on the basal level of ERK1/2 phosphorylation. Interestingly however, although the knockdown of βarr1 or 2 did not affect the elevated basal level of ERK1/2 phosphorylation in C5aR2-expressing cells, βarr2 depletion appears to reduce the effect of C5a on lowering ERK1/2 phosphorylation (Figures S3D, S3E, and S4H).

### Phospho-antibody array and MS-based phosphoproteomics analysis for D6R

In order to identify potential pathways involved in signaling downstream of D6R, we used a Phospho Explorer Antibody Array (Full Moon Biosystems) designed for broad-scope protein phosphorylation profiling. This array consists of 1,318 different antibodies related to multiple signaling pathways and biological processes and, therefore, allowed us to measure the change in the phosphorylation status of a large set of proteins upon activation of D6R. In order to specifically monitor D6R-activation-dependent cellular proteins, we compared the cellular lysates prepared under the basal and agonist-treatment conditions (Figure S5A). A list of all the target proteins, their phosphorylation sites being detected using this array, and the fold change over basal (agonist treatment versus no treatment) are presented in Table S1. We observed that about 30 target proteins exhibited a ≥ 1.4-fold increase in their phosphorylation upon agonist stimulation over the basal condition, whereas another 30 proteins displayed a ≤ 0.75-fold decrease in their phosphorylation status (Figure S5C; Table S1).

**Figure 6:**
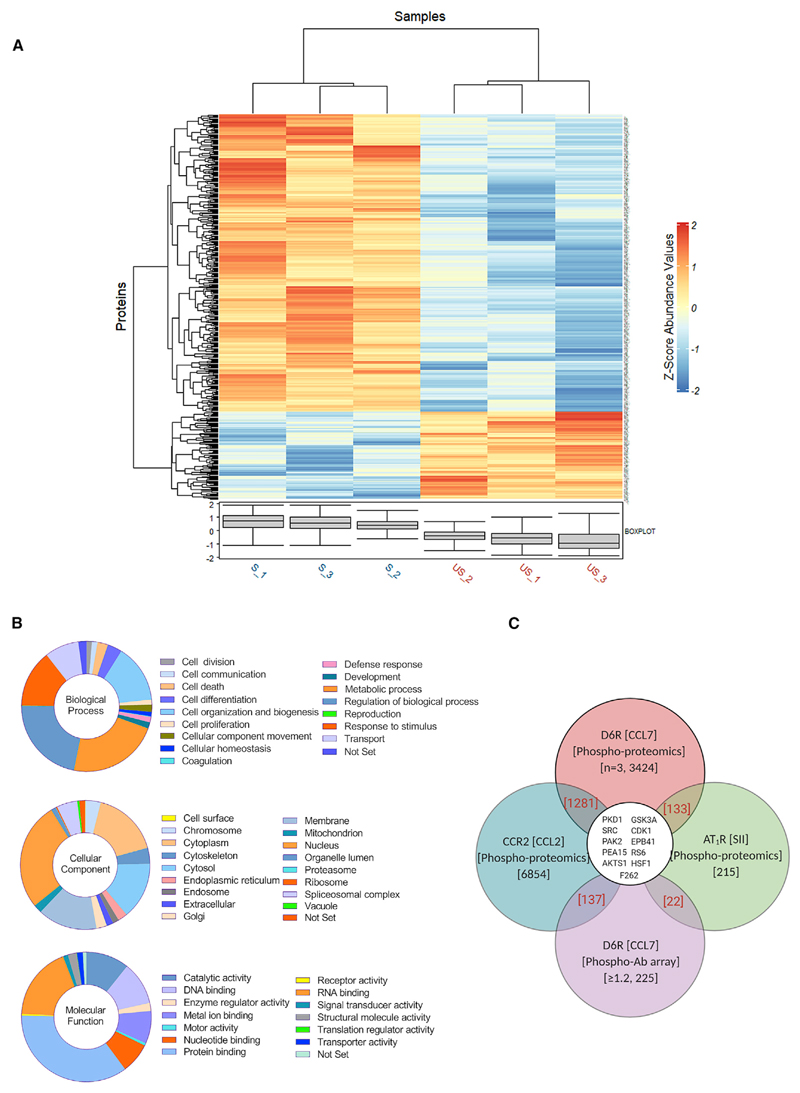
Phospho-proteomics reveals potential D6R signaling pathways (A) Heatmap depicting differentially phosphorylated proteins generated based on phospho-proteomics analysis on HEK293 cells expressing D6R with and without agonist stimulation. Three independent lysate samples prepared in parallel were used for trypsin digestion, enrichment of phospho-peptides using TiO_2_, and mass spectrometry (MS)-based identification of cellular proteins. S1–S3 indicate replicates of stimulated samples, while US1–US3 indicate replicates of unstimulated samples. (B) Classification of cellular proteins that undergo phosphorylation/dephosphorylation upon D6R stimulation based on biological processes, molecular functions, and cellular localization reveal an extensive network of potential signaling pathways. (C) Comparison of D6R phospho-proteomics data with phospho-antibody array and previously published phospho-proteomics study on CCR2 and AT1R reveals the activation of some common and multiple D6R-specific signaling proteins. Details of the common proteins are listed in Figure S6F. See also Figures S5–S7 and Tables S1 and S2.

We also carried out a mass-spectrometry (MS)-based phosphoproteomics study using HEK293 cells expressing D6R and compared the samples prepared under basal and stimulated conditions (Figure 6A; Figure S5B). We generated the proteolytic fragments by using trypsin digestion of cellular lysate from both conditions followed by TiO_2_-based phospho-peptide enrichment and detection of phospho-peptides by using liquid chromatography-tandem MS (LC-MS/MS). A simplified schematic of the phospho-proteomics experiment is described in Figure S5B, and the complete details of data including the identified hits, fold change in phosphorylation status, and other parameters are included in Table S2. We identified a total of 2,220 proteins, of which 1,265 were phospho-proteins, i.e., 57% of the identified proteins after TiO_2_-based phospho-enrichment were phospho-proteins. The total number of identified peptides was 4,972, of which 3,424 were phospho-peptides, i.e., 69% of the identified peptides were phospho-peptides. Based on t test statistics on the two groups, i.e., basal and stimulated, we identified a total of 444 significant phospho-peptides, which exhibited differential abundance between the 2 conditions (Figure S6A).

A classification of these proteins based on their cellular localization, molecular function, and biological processes suggests that D6R activation is linked to a broad spectrum of cellular and functional outcomes (Figure 6B). In order to gain further in-sights into D6R signaling, we first compared the phospho-proteins identified in the Phospho Explorer Antibody Array and MS-based phospho-proteomics study by manually curating and analyzing the hits obtained with these two methods. We identified 46 different proteins that were common to both datasets (Figure S6B). Next, we compared the phospho-proteins identified in the Phospho Explorer Antibody Array and MS-based phosphoproteomics study with those described previously in the context of either βarr-biased agonism or for another chemokine receptor. These previously published datasets include three different studies measuring the phospho-proteins upon stimulation of the angiotensin II subtype 1 receptor (AT1R) by using a βarr-biased agonist SII (Christensen et al., 2010; Kendall et al., 2011; Xiao et al., 2010) and a recent study describing the phospho-proteome of another chemokine receptor, CCR2, in response to stimulation with the CCL2 chemokine (Huang et al., 2020a). The notion for this comparison is 2-fold—first, to identify potentially conserved downstream signaling proteins involved in βarr-mediated signaling, and second, to assess the phospho-proteins that are not present in other datasets and, therefore, are likely to be D6R specific. This comparison was also performed by manual curation and analysis by using our dataset and a previously published dataset. This comparison identified not only a number of common proteins present in these studies but also several proteins that are specific to D6R activation (Figure 6C; Figures S6C–S6E). These findings underline that some of the signaling downstream of D6R may be potentially similar to that identified for other GPCRs in the context of βarr-mediated pathways, while there may also exist receptor-specific and previously unidentified pathways downstream of D6R. It should be noted here that SII elicits measurable Gαi and Gα12 signaling responses (Namkung et al., 2018), and therefore, a part of the hits identified earlier may not be exclusively βarr dependent.

We also experimentally validated the phosphorylation of three different proteins, namely, cofilin (Ser^3^), the platelet-derived growth factor receptor (PDGFR-β) (Tyr^751^), and protein kinase D (PKD) (Ser^744/748^), upon CCL7 stimulation in D6R-expressing cells, and we observed an agonist-induced, time-dependent response (Figures S7A–S7C). We also observed that agonist-induced phosphorylation of PDGFR-β (Tyr^751^) (Figure S7D) and cofilin (Ser^3^) (Figure S7E) is significantly attenuated upon βarr knockdown, suggesting a direct involvement of βarrs. Interestingly, a previous study has reported that the phosphorylation of cofilin upon stimulation of D6R with another chemokine, CCL2, is also reduced by βarr1 depletion (Borroni et al., 2013). Taken together, these data suggest a broad signaling network downstream of D6R and set the stage for further investigation of specific signaling pathways and corresponding cellular outcomes.

### C5aR2 activation leads to p90RSK phosphorylation and neutrophil mobilization

**Figure 7:**
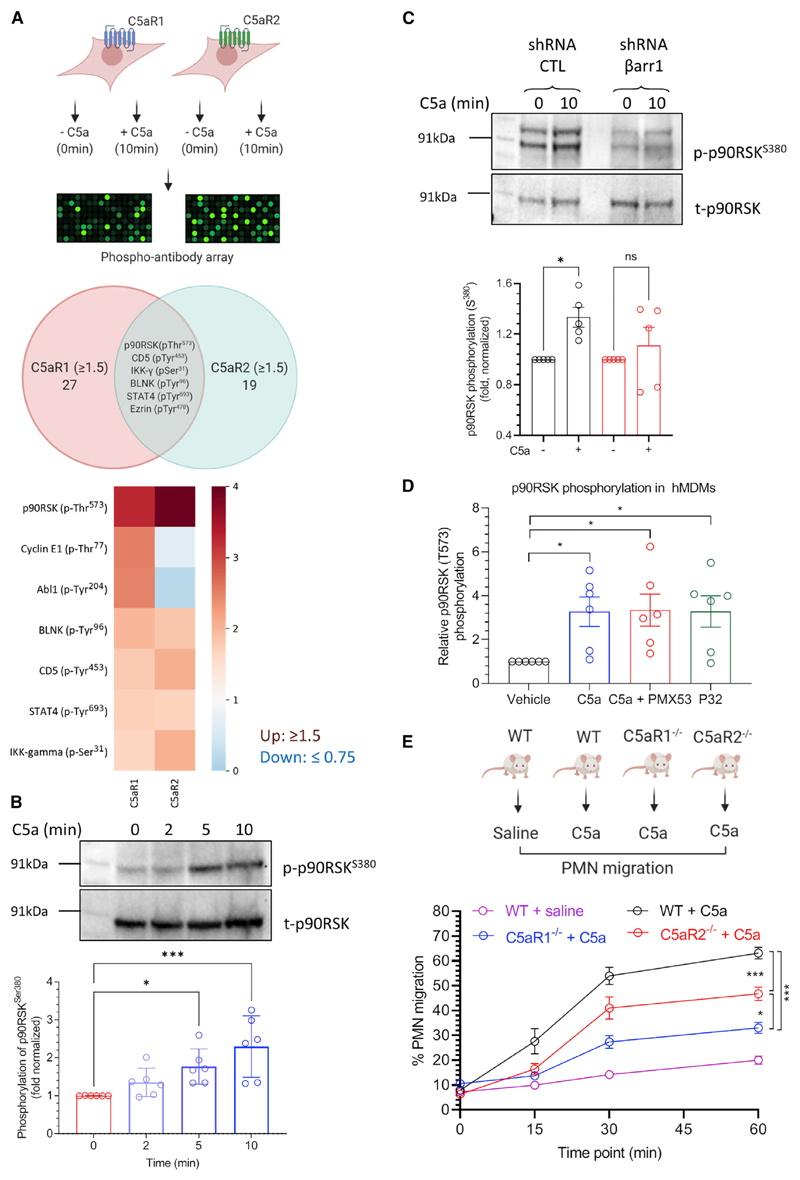
Signaling and functional outcomes of C5aR2 activation (A) Phospho-antibody array on HEK293 cells expressing C5aR1 or C5aR2 reveals phosphorylation/dephosphorylation of a few common and several distinct cellular proteins. Of these proteins, C5a stimulation of C5aR2-expressing HEK293 cells exhibits robust enhancement of p90RSK phosphorylation, which is also common to C5aR1. (B) C5a-induced phosphorylation of p90RSK^Ser380^ in C5aR2-expressing HEK293 cells is validated using western blotting. A representative blot and densitometry-based quantification (mean ± SEM) from six independent experiments are presented (*p < 0.05, ***p < 0.001; one-way ANOVA). (C) C5a-induced phosphorylation of p90RSK^Ser380^ is attenuated upon βarr1 knockdown in HEK293 cells expressing C5aR2. A representative blot and densitometry-based quantification (mean ± SEM) from five independent experiments are presented (*p < 0.05; one-way ANOVA). (D) Stimulation of human-macrophage-derived monocytes (hMDMs) with either C5a or P32 (a C5aR2-selective agonist) results in phosphorylation of p90RSK^Thr573^. Importantly, PMX53, a C5aR1-selective antagonist, does not block p90RSK^Thr573^ phosphorylation, suggesting that it is mediated by C5aR2. (E) A significant component of C5a-induced polymorphonuclear leukocyte (PMN) mobilization depends on C5aR2. Wild-type (WT) and C5aR1^−/−^ and C5aR2^−/−^ mice on a C57BL/6J genetic background (n = 5–15) were intravenously administered with recombinant mouse C5a (50 μg kg^−1^). Blood collected from the tail tip was smeared onto a slide, followed by staining and counting of white blood cells, with the proportion of PMNs calculated. Data are presented as mean ± SEM (p < 0.05, ***p < 0.001; one- and two-way ANOVA). See also Figure S7 and Table S1.

In order to probe if C5aR2 may signal through non-canonical pathways, we carried out a phospho-antibody-array-based screen to identify cellular proteins that undergo a change in their phosphorylation level upon C5aR1 and C5aR2 stimulation, similar to that described for D6R above. We observed that a number of proteins undergo phosphorylation/dephosphorylation upon stimulation of C5aR1- and C5aR2-expressing cells (Table S1), and interestingly, several of the proteins were common to both receptors (Figure 7A), suggesting a potential involvement of βarrs. We experimentally validated agonist-induced phosphorylation of one of these proteins, p90RSK, at three different phosphorylation sites, namely, Thr^359^, Ser^380^, and Thr^573^. We observed agonist-induced and time-dependent phosphorylation at Ser^380^ (Figure 7B), whereas the other two sites did not yield consistent data. Importantly, C5a-induced phosphorylation of p90RSK at Ser^380^ is reduced upon βarr1 knockdown in HEK293 cells, suggesting an involvement of βarr1 (Figure 7C).

In order to further corroborate this finding, we measured C5a-induced p90RSK phosphorylation in human-monocyte-derived macrophages (HMDMs) by using an in-cell ELISA approach. These cells constitutively express both of the receptors, i.e., C5aR1 and C5aR2, although efficient knockdown of βarrs in these cells has technical limitations. Therefore, we used a pharmacological approach to dissect the specific contribution of C5aR2 by using a C5aR2-specific agonist (P32) (Croker et al., 2016). As presented in Figure 7D, we found that C5a-induced p90RSK phosphorylation at Thr^573^ in HMDMs was identical to that induced by P32. Interestingly, pre-treatment of these cells with a C5aR1-specific antagonist (PMX53) (Li et al., 2020b) did not block P32-induced p90RSK phosphorylation, suggesting a direct involvement of C5aR2 (Figure 7D). On the other hand, C5a-induced phosphorylation of Thr^359^ and Ser^380^ in HMDMs appears to be mediated primarily by C5aR1, as pre-treatment with PMX53 blocks C5a response and stimulation with P32 does not yield a significant response (Figures S7F and S7G).

In order to identify a potential cellular and physiologically relevant effect mediated by C5aR2, we used C5aR1 and C5aR2 knockout mice to measure C5a-induced polymorphonuclear leukocyte (PMN) mobilization to the blood. We observed that intravenous C5a administration induced robust PMN mobilization in wild-type mice in a time-dependent manner, which was significantly reduced but not completely abolished by C5aR1 knockout (Figure 7E). Notably, PMN mobilization is also reduced in C5aR2 knockout mice, albeit at lower levels than C5aR1, suggesting a distinct role of C5aR2 in PMN mobilization, in addition to the major role played by C5aR1 (Figure 7E). Taken together, these data suggest a direct contribution of C5aR2 activation in the mobilization of granulocytes into the bloodstream following a C5a signal and establish a distinct functional response elicited through this receptor *in vivo*.

## Discussion

Although D6R and C5aR2 do not activate G proteins, an interesting question that remains is whether they lack a physical interaction with G proteins or an ability to activate G proteins despite a physical interaction. It would also be tantalizing to explore whether D6R and C5aR2 undergo an activation-dependent conformational change similar to that observed for prototypical GPCRs, including outward movement of transmembrane (TM) helix 5 and 6 (Weis and Kobilka, 2018). It is plausible that activation-dependent outward movement of TM5 and 6 is restricted in these receptors, which does not permit the G protein interaction, although additional studies are required to test this possibility. Another intriguing observation is the constitutive phosphorylation of D6R, which does not change significantly upon CCL7 stimulation. Although we observe some level of constitutive localization of βarrs in the membrane for D6R, it is significantly enhanced upon CCL7 stimulation, followed by distribution in endosomal vesicles. Taken together with the βarr-recruitment profile in GRK knockout cells and for truncated receptor constructs, it is possible that receptor phosphorylation has a rather minor contribution in βarr recruitment for D6R. These findings potentially uncover a non-canonical mode of interaction between a 7TMR and βarrs without a major role of receptor phosphorylation, which is considered as a generic paradigm in the 7TMR family. Some earlier studies have suggested a potential contribution of negatively charged residues in phosphorylation-independent βarr recruitment to GPCRs (Mukherjee et al., 2002; Zhou et al., 2017), and our findings with D6R may catalyze the discovery of additional such examples.

C5aR2 has also been an enigmatic receptor since its discovery due to a lack of G protein activation, and it has been shown to be involved in broadly modulating GPCR signaling including that of C5aR1 (Croker et al., 2014; Li et al., 2020a). However, the activation of a signaling pathway directly downstream of C5aR2 has not been established yet. We observe an elevated level of basal ERK1/2 phosphorylation in HEK293 cells expressing C5aR2, which is reduced upon C5a stimulation, but it does not appear to involve Gαi or βarrs. Importantly, we also discover that several cellular proteins undergo a change in their phosphorylation status upon C5aR2 activation and that p90RSK phosphorylation downstream of C5aR2 is sensitive to βarr1 depletion. Therefore, our study provides a framework for exploring additional signaling pathways downstream of C5aR2 and may help uncover additional functions of C5aR2 going forward. It is important to note that although C5a is typically considered the shared endogenous agonist for C5aR1 and C5aR2, it cannot be completely ruled out that it may be a βarr-biased ligand at C5aR2, and balanced C5aR2 agonists are yet to be discovered. The interplay of G proteins and βarrs in ERK1/2 MAP kinase activation downstream of GPCRs has emerged as an interesting paradigm recently (Dwivedi et al., 2018; Grundmann et al., 2018; Luttrell et al., 2018; O’Hayre et al., 2017; Wang et al., 2017). In this context, our data underscore two interesting points. First, recruitment of βarrs to 7TMRs may not necessarily translate to ERK1/2 activation, and second, there may exist receptor-specific fine-tuning of this interplay. Our findings establish D6R and C5aR2, together with their prototypical GPCR counterparts, as an interesting system to further probe the mechanistic interplay of G proteins and βarrs in ERK1/2 activation.

Distinct binding modes and conformations of βarrs have emerged as primary mechanisms driving their multifunctionality and functional diversity (Cahill et al., 2017; Gurevich and Gurevich, 2006; Kumari et al., 2016, 2017). Recent structures of GPCR-arrestin complexes have also revealed the distinct orientation of arrestins in complex with different receptors (Huang et al., 2020b; Kang et al., 2015; Lee et al., 2020; Staus et al., 2020). For example, a comparison of the rhodopsin-visual-arrestin structure with the neurotensin receptor-βarr1 complex reveals a large rotation of βarr1 in the plane of the membrane, which is not apparent in the M2R-βarr1 and β1AR-βarr1 structures (Chaturvedi et al., 2020). Distinct conformational signatures of βarrs upon their interaction with D6R and C5aR2 compared to CCR2 and C5aR1 observed in the current study further underscore the conformational diversity in the 7TMR-βarr interaction. Previous studies have linked distinct βarr conformations to different functional outcomes, such as desensitization, endocytosis, and signaling, although a clean separation of these functional outcomes has been technically challenging (Cahill et al., 2017; Kumari et al., 2016, 2017; Shukla et al., 2008; Zimmerman et al., 2012). Our study now provides an additional handle in the form of these βarr-coupled 7TM receptors to decipher and link conformational signatures in βarrs to specific functional outcomes.

In summary, our study establishes D6R and C5aR2 as “arrestin-coupled receptors” with a lack of detectable G protein coupling and potential signaling through non-canonical pathways. Moreover, we also establish that βarrs adopt distinct conformations upon interaction with these receptors compared to their prototypical GPCR counterparts, which highlights the conformational diversity of 7TMR-βarr complexes. Ourf indings underscore distinct functional capabilities of 7TMRs, and they have broad implications to better understand the framework of biased agonism at these receptors.

## Limitations of the Study

We note that several experiments in this study are carried out under receptor overexpression conditions, although we have maintained a moderate level of receptor expression and validated the key findings in primary cells. Importantly, however, we have monitored agonist-induced responses in every experiment and, therefore, measured values likely represent receptor-mediated and receptor-activation-dependent outcomes. We also note that in the phospho-proteomics experiment, we used one-step TiO_2_-based enrichment of phospho-peptides but did not perform additional fractionation steps, which could have further increased the number of phospho-peptides.

## STAR⋆Methods

### Key Resources Table

**Table:** 

REAGENT or RESOURCE	SOURCE	IDENTIFIER
Antibodies		
Monoclonal ANTI-FLAG M2-HRP antibody	Sigma-Aldrich	Cat# A8592; RRID:AB_439702
Phospho-p44/42 MAPK (Erk1/2)	Cell Signaling Technology	Cat# 9101; RRID:AB_331646
p44/42 MAPK (Erk1/2) Antibody	Cell Signaling Technology	Cat# 9102; RRID:AB_330744
β-Arrestin 1/2 (D24H9) Rabbit mAb	Cell Signaling Technology	Cat# 4674; RRID:AB_10547883
Anti-phospho-Cofilin Ser^3^ antibody	Cell Signaling Technology	Cat# 3313; RRID:AB _2080597
Anti-phospho-PDGFR Tyr^751^ antibody	Cell Signaling Technology	Cat# 4549; RRID:AB_1147704
Anti-phospho-PKD Ser^744/748^ antibody	Cell Signaling Technology	Cat# 2054; RRID:AB_2172539
Phospho-P90RSK (Ser^380^)(D3H11) Rabbit mAb	Cell Signaling Technology	Cat# 11989; RRID:AB_2687613
Phospho-P90RSK (Thr359)(D1E9) Rabbit mAb	Cell Signaling Technology	Cat# 8753; RRID:AB_2783561
Phospho-P90RSK (Thr573) Antibody	Cell Signaling Technology	Cat# 9346; RRID:AB_330795
IRDye 680RD donkey anti-rabbit secondary Ab	LI-COR Biosciences	Cat# 926-32212; RRID:AB_621847
Anti-FLAG M1 antibody (1:100) - DyLight594	In-house	N/A
RSK1/RSK2/RSK3 (32D7) Rabbit mAb	Cell Signaling Technology	Cat# 9355; RRID:AB_659900
Anti-b actin antibody	Sigma	Cat# A3854; RRID:AB_262011
Anti-rabbit IgG secondary antibody	GenScript	Cat# A00098; RRID:AB_1968815
Anti-FLAG epitope tag monoclonal antibody	FujiFilm Wako Pure Chem	Cat# 012-22384; RRID: AB 10659717
Chemicals, peptides, and recombinant proteins		
DSP (Dithiobis succinimidyl-propionate)	Sigma Aldrich	Cat# D3669
Paraformaldehyde (PFA)	Sigma Aldrich	Cat# P6148, CAS no. 30525-89-4
Poly-D-lysine	Sigma Aldrich	Cat# P0899
Poly-L-Ornithine Solution	Sigma Aldrich	Cat# P2533
Phenylmethane Sulphonyl Fluoride (PMSF)	SRL	Cat# 84375 (84375)
Benzamidine Hydrochloride	SRL	Cat# 93014 (0248255)
Phosphatase inhibitor cocktail (Phosstop)	Roche	Cat# 4906837001
Lauryl Maltose Neopentyl Glycol (MNG)	Anatrace	Cat# NG310, CAS no.1257852-96-2
NP-40	Sigma Aldrich	Cat# 492016, CAS no. 9016-45-9
FLAG peptide	Genscript	N/A
TMB (Tetramethylbenzidine)	Thermo Fisher Scientific	Cat# 34028
Janus Green B	Sigma Aldrich	Cat# 201677
Luciferin sodium salt	Gold Biotech	Cat# LUCNA, CAS no. 103404-75-7
Puromycin dihydrochloride	Gold Biotech	Cat# P-600-100
Geneticin™ Selective Antibiotic	GIBCO	Cat# 11811-031
PEI (Polyethylenimine)	Polysciences	Cat# 23966
Bovine Serum Albumin, BSA	SRL	Cat# 83803 (0140105)
HBSS - Hank’s Balanced Salt Solution	Thermo Fisher Scientific	Cat# 14065
Forskolin	Sigma Aldrich	Cat# F6886
GIBCO Fetal Bovine Serum	Thermo Fisher Scientific	Cat# 10270-106
DMEM	Cellclone	Cat# CC3004
Phosphate-buffered saline (PBS)	Sigma Aldrich	Cat# D1283
GIBCO Penicillin-Streptomycin	Thermo Fisher Scientific	Cat# 15140122
ESF 921 Insect Cell Culture Medium	Expression Systems	Cat# 96-001-01
Pertussis	Toxin Apex Bio	Cat# B7273
Coelenterazine	Carbosynth/Goldbio	Cat# EC14031/Cat# CZ05
Coelenterazine H	NanoLight Technology	Cat# 301-500
Fluo4-NW dye	Invitrogen	Cat# F36206
Triton X-100	Sigma Aldrich	Cat# 9002-93-1
DAPI stain	Sigma Aldrich	Cat# D9542
VectaShield HardSet mounting medium	VectaShield	Cat# H-1400
ECL solution	Promega	Cat# W1015
Pan MEK inhibitor U0126	Sigma Aldrich	Cat# 19-147
Recombinant human C5a	Purified	N/A
Recombinant human CCL7	Purified	N/A
Bradykinin	Genscript	N/A
Arginine Vasopressin Peptide (AVP)	Genscript	N/A
PMX53	Synthesized (in-house)	N/A
W54011	Tocris	Cat# 5455
P32	Synthesized (in-house)	NA
Hemacolor® Solution 2 (Eosin Y) and Hemacolor® Solution 3 (Azur B)	Merck KGaA, 64271 Darmstadt, Germany	Cat# 111661
Uranyl formate	Polysciences	Cat# 24762-1
Formvar/carbon coated 300 mesh copper grids	PELCO (Ted Pella)	Cat# 01753-F
BCA/Copper solution	G Bioscience	Cat# 786-845
4-20% precast gradient gel	Bio-Rad	Cat# 4561-093
Critical commercial assays		
Glosensor	Promega	N/A
pIMAGO-biotin Phosphoprotein detection kit	Sigma Aldrich	Cat# 18419
Site directed mutagenesis kit	NEB	Cat# E0554
Fullmoon Biosystems for phospho-antibody array	Full Moon BioSystems	Cat# KAS02
NanoBiT assay	Promega	N/A
Deposited data		
Gel images and confocal images	This study	Mendeley data https://doi.org/10.17632/nzpd6k32gz.1
Phosphoproteomics data	This study	ProteomeXchange PXD027887
Experimental models: Cell lines		
Human: HEK293	ATCC	Cat# CRL-3216
Human: HEK293A	Thermo Fisher Scientific	Cat# R70507
Human: HEK293SL	Stephane A. Laporte Asuka Inoue	N/A
ΔGRK2/3 HEK293A cells	Asuka Inoue	N/A
ΔGRK5/6 HEK293A cells	Asuka Inoue	N/A
ΔGRK2/3/5/6 HEK293A cells	Asuka Inoue	N/A
Insect: Sf9	Expression Systems	94-001F
Oligonucleotides		
D6R^Δ351^ C tail truncation SDM primers Forward: TAAATACTTACTG CCCAAGAGG	This study	N/A
D6R^Δ351^ C tail truncation SDM primers Reverse: GCTGCTCTCAGAACAGCT	This study	N/A
D6R^Δ342^ C tail truncation SDM primers Forward: TAATCATTATCCAGCTGTTCTGAGAGCAGCATAC	This study	N/A
D6R^Δ342^ C tail truncation SDM primers Reverse: GGCCTGGGCAGTGCCAGG	This study	N/A
Recombinant DNA		
pcDNA_βarr1/2-mcherry	Dr Mark G.H.Scott	N/A
pLKO.1_βarr1/2 shRNA	Dr Hyder Ali	N/A
pcDNA_βarr1-mYFP	Addgene	Plasmid #36916
pcDNA_βarr2-mYFP	Addgene	Plasmid #36917
pCMV-AC6_βarr1	Dr Arun K Shukla	N/A
pCMV-AC6_βarr2	Dr Arun K Shukla	N/A
pcDNA_R-Luc-βarr2-FlAsH	Dr Stephane Laporte	N/A
pCMV-AC6-Ib30-YFP	Dr Arun K Shukla	N/A
pcDNA-FLAG-C5aR1-WT	Dr Arun K Shukla	N/A
pcDNA-FLAG-C5aR2-WT	This paper	N/A
pcDNA-FLAG-CCR2-WT	This paper	N/A
pcDNA-FLAG-D6R-WT	Dr Arun K Shukla	N/A
pcDNA-FLAG-D6R^Δ351^	This paper	N/A
pcDNA-FLAG-D6R^Δ342^	This paper	N/A
pvL1393-FLAG-C5aR2	This paper	N/A
pvL1393-βarr1	This paper	N/A
pvL1393-GRK6	Dr Arun K Shukla	N/A
Software and algorithms		
Graphpad Prism 8	Graphpad	N/A
Zen lite, Zeiss	Zeiss	https://www.zeiss.com/microscopy/int/products/microscope-software/zen-lite.html
EMAN2.31	Tang et al., 2007	https://blake.bcm.edu/emanwiki/EMAN2
ISAC2	Yang et al., 2012	https://sphire.mpg.de/wiki/doku.php?id=pipeline:isac:sxisac2
SPHIRE	Moriya et al., 2017	https://sphire.mpg.de/wiki/doku.php
ImageJ	(Schneider et al., 2012)	https://imagej.nih.gov/ij/download.html
KEA2.0	(Lachmann and Ma'ayan, 2009)	https://www.maayanlab.net/KEA2/
KEA3	(Kuleshov et al., 2021)	https://maayanlab.cloud/kea3
Other		
M1-FLAG resin	In-house	N/A
CaptoL (Protein L)	GE Lifesciences	Cat# 17547802
Mouse anti-HA (Hemagglutinin) resin	Sigma	Cat# A2095

### Resource Availability

#### Lead contact

Further information and requests for resources and reagents should be directed to the Lead Contact, Dr. Arun K. Shukla (arshukla@iitk.ac.in).

#### Materials availability

Plasmids described in this paper are available from the lead contact upon reasonable request and MTA.

### Experimental Models and Subject Details

#### Human cell lines

HEK293 cells were obtained from ATCC and HEK293A cells were purchased from Thermo Fisher Scientific. Cell lines were frequently checked for proper morphology under the microscope but were not authenticated. These cell lines were cultured in DMEM with 10% fetal bovine serum (FBS) at 37°C in 5% CO_2_ (v/v). Any new stable, knock-out and knock-down cell lines were not generated in this study, and the details of the previously generated cell lines used here are mentioned and referenced in the text.

#### Insect cells

The *Sf9* cells were purchased from Expression Systems and were routinely monitored for morphology under the microscope. These cells were sub-cultured in protein free insect cell media purchased from Expression Systems, and maintained at 27°C with 135 rpm shaking in a shaker incubator.

### Method Details

#### General chemicals and reagents

Most of the general chemicals and molecular biology reagents were purchased from Sigma unless mentioned otherwise. HEK293 cells (ATCC) were maintained at 37°C under 5% CO_2_ in Dulbecco’s modified Eagle’s medium (GIBCO, Cat. no. 12800-017) supplemented with 10% FBS (GIBCO, Cat. no. 10270-106) and 100 U ml^-1^ penicillin and 100 μg ml^-1^ streptomycin (GIBCO, Cat. no. 15140-122). Stable cell lines expressing N-terminal Flag tagged receptor constructs in pcDNA3.1 vector were generated by transfecting HEK293 cells with 7 μg of plasmid DNA using polyethylenimine (PEI) (Polysciences, Cat. no. 19850), followed by selection using G418 (GIBCO, Cat. no. 11811-031, 200-1000 μg ml^-1^). Single cell clones, which survived G418 selection, were subsequently expanded and sub-cultured. βarr1 and βarr2 shRNA expressing stable cell lines have been described earlier (Ghosh et al., 2019), and they were cultured in DMEM containing 10% (FBS), 100 U ml^-1^ penicillin and 100 μg ml^-1^ streptomycin, and 1.5μg ml^-1^ puromycin dihydrochloride (GoldBio, Cat. no. P-600). Recombinant C5a (human) and CCL7 (human) ligands were expressed and purified as described previously (Goncharuk et al., 2020; Pandey et al., 2019a). The plasmids encoding FLAG–C5aR1, FLAG–C5aR2, FLAG–CCR2, FLAG-D6R, βarr1–mCherry, βarr1–mYFP, βarr2-mYFP, Ib30-YFP have been described previously (Baidya et al., 2020b; Ghosh et al., 2017, 2019; Kumari et al., 2016; Pandey et al., 2019a). All constructs were verified by DNA sequencing (Macrogen). The antibodies used in this study were purchased either from Sigma (HRP-coupled mouse anti-FLAG M2, HRP-coupled anti-β-actin, HRP-coupled anti-rabbit) or from Cell Signaling Technology (βarrs, ERK, PKD, PDGFRB, Cofilin, P90RSK).

#### NanoBiT-based G protein dissociation assay

Agonist-induced G protein activation was measured by a NanoBiT-based G protein dissociation assay (Inoue et al., 2019), in which dissociation of a Gα subunit from a Gβγ subunit was monitored by a NanoBiT system (Promega). Specifically, a NanoBiT-G-protein consisting of a large fragment (LgBiT)-containing Gα subunit and a small fragment (SmBiT)-fused Gγ_2_ subunit with a C68S mutation, along with the untagged Gβ_1_ subunit, was expressed with a test GPCR construct, and the ligand-induced luminescent signal change was measured. HEK293A cells (Thermo Fisher Scientific) were seeded in a 6-well culture plate (Greiner Bio-One) at a concentration of 2 × 10^5^ cells ml^-1^ (2 mL per dish hereafter) in DMEM (Nissui Pharmaceutical) supplemented with 10% FBS (GIBCO), glutamine, penicillin, and streptomycin, one day before transfection. The transfection solution was prepared by combining 5 μl of polyethylenimine solution (1 mg ml^-1^) and a plasmid mixture consisting of 100 ng LgBiT-containing Gα, 500 ng Gβ_1_, 500 ng SmBiT-fused Gγ_2_ (C68S), and an indicated volume (below) of a test GPCR with N-terminal HA-derived signal sequence and FLAG-epitope tag followed by a flexible linker (MKTIIALSYIFCLVFADYKDDDDKGGSGGGGSGGSSSGGG; ssHA-FLAG-GPCR) in 200 μl of Opti-MEM (Thermo Fisher Scientific). To measure dissociation of the other G protein families, we used LgBiT-Gα_i1_ subunit plasmid (G_i1_), LgBiT-Gα_i2_ subunit plasmid (G_i2_), LgBiT-Gα_i3_ subunit plasmid (G_i3_), LgBiT-Gα_o_ subunit plasmid (G_o_), LgBiT-Gα_s_ subunit (G_s_), LgBiT-Gα_q_ subunit (G_q_), LgBiT-Gα_12_ subunit (G_12_) and LgBiT-Gα_13_ subunit (G_13_). To enhance NanoBiT-G-protein expression for G_s_, G_q_ and G_13_, 100 ng plasmid of RIC8B (isoform 2; for G_s_) or RIC8A (isoform 2; for G_q_, G_12_, and G_13_) was additionally co-transfected. To match the expression of the receptor pairs, 40 ng (C5aR1; with 160 ng of an empty vector) and 200 ng (C5aR2, CCR2 and D6R) plasmids were used. After an incubation for one day, the transfected cells were harvested with 0.5 mM EDTA-containing Dulbecco’s PBS, centrifuged, and suspended in 2 mL of Hank’s balanced saline solution (HBSS, GIBCO, Cat. no. 14065-056) containing 0.01% bovine serum albumin (BSA fatty acid-free grade, SERVA) and 5 mM HEPES (pH 7.4) (assay buffer). The cell suspension was dispensed in a white 96-well plate at a volume of 80 μl per well and loaded with 20 μl of 50 μM coelenterazine (Carbosynth), diluted in the assay buffer. After 2 h incubation, the plate was measured for baseline luminescence (SpectraMax L, Molecular Devices) and 20 μl of 6X test compound (C5a or CCL7), serially diluted in the assay buffer, were manually added. The plate was immediately read for the second measurement as a kinetics mode and luminescence counts recorded from 3 min to 5 min after compound addition were averaged and normalized to the initial counts. The fold-change signals were further normalized to the vehicle-treated signal and were plotted as a G protein dissociation response. Using the Prism 8 software (GraphPad Prism), the G protein dissociation signals were fitted to a four-parameter sigmoidal concentration-response curve.

#### Receptor surface expression assay

For measuring surface expression of the receptors, whole-cell based surface ELISA was performed as described previously (Pandey et al., 2019b). Briefly, receptor-expressing cells were seeded in 24-well plate (pre-coated with poly-D-Lysine) at a density of 0.1 million per well. After 24 h, media was removed, and cells were washed once with ice-cold 1XTBS followed by fixation with 4% PFA (w/v in 1XTBS) on ice for 20 min and subsequent extensive washing with 1XTBS. Blocking was done with 1% BSA prepared in 1XTBS for 1.5 h, which was followed by incubation of cells anti-FLAG M2-HRP antibody (Sigma, Cat no. A8592) at a dilution of 1:2000 prepared in 1% BSA+1XTBS for 1.5 h. Subsequently, cells were washed thrice with 1% BSA (in 1XTBS) to rinse off any unbound traces of antibody. Cells were incubated with 200 μL of TMB-ELISA (Thermo Scientific, Cat. no: 34028) substrate till the appearance of a light blue color and reaction was stopped by transferring 100 μL of this solution to a different 96-well plate containing 100 μL of 1 M H_2_SO_4_. Absorbance was recorded at 450 nm in a multi-mode plate reader (Victor X4, Perkin Elmer). For normalization of signal intensity, cell density was estimated using a mitochondrial stain Janus green B. Briefly, TMB was removed and cells were washed twice with 1XTBS followed by incubation with 0.2% Janus green B (Sigma, Cat. no. 201677) (w/v) for 15 min. Cells were destained by extensively washing with distilled water. The stain was eluted by adding 800 μLof 0.5 N HCl per well. 200 μLof this solution was transferred to a 96-well plate and absorbance was read at 595 nm. Data normalization was performed by calculating the ratio of A_450_ to A_595_ values.

In the NanoBiT assays, surface expression was measured using flow-cytometry based assay. Briefly, HEK293A cells were transfected as described in the “NanoBiT-based G protein dissociation assay” section. One day after transfection, the cells were harvested with 0.5 mM EDTA-containing Dulbecco’s PBS (D-PBS). Forty percent of the cell suspension was transferred in a 96-well V-bottom plate and fluorescently labeled by using anti-FLAG epitope (DYKDDDDK) tag monoclonal antibody (Clone 1E6, FujiFilm Wako Pure Chemicals; 10 μg ml^-1^ diluted in 2% goat serum- and 2 mM EDTA-containing D-PBS (blocking buffer)) and a goat anti-mouse IgG secondary antibody conjugated with Alexa Fluor 488 (Thermo Fisher Scientific; 10 mg ml^-1^ in diluted in the blocking buffer). After washing with D-PBS, the cells were resuspended in 200 μL of 2 mM EDTA-containing D-PBS and filtered through a 40 μm filter. Fluorescent intensity of single cells was quantified by an EC800 flow cytometer equipped with a 488 nm laser (Sony). Fluorescent signal derived from Alexa Fluor 488 was recorded in a FL1 channel and flow cytometry data were analyzed by a FlowJo software (FlowJo). Live cells were gated with a forward scatter (FS-Peak-Lin) cutoff of 390 setting a gain value of 1.7. Values of mean fluorescence intensity (MFI) from approximately 20,000 cells per sample were used for analysis.

#### cAMP assay

Ligand-induced Gαs- and Gαi-activation was assessed by measuring cAMP with Glosensor assay as described previously (Kumari et al., 2017). Briefly, HEK293 cells were transfected with FLAG-tagged receptor (3.5 μg) and luciferase-based 22F cAMP biosensor construct (3.5 μg) (Promega). 14–16 h post transfection, cells were harvested and resuspended in assay buffer containing D-luciferin (0.5 mg ml^-1^, GoldBio, Cat. no. LUCNA-1G) in 1X HBSS, pH 7.4 and 20 mM of 4-(2-hydroxyethyl)-1-piperazineethanesulfonic acid (HEPES). Cells were seeded in 96 well white plates (Corning) at a density of 125,000 cells per 100 μL and incubated at 37°C for 90 min. This was followed by an additional incubation of 30 min at room temperature. For stimulation, ligand doses (AVP for V_2_R, C5a for C5aR1 and C5aR2; CCL7 for D6R) were prepared by serial dilution ranging from 0.1 pM to 1 μM and were added to respective wells. For Gαi activation assay, prior to ligand addition, cells were treated with forskolin (5 μM). Luminescence was recorded using a microplate reader (Victor X4; Perkin Elmer). Data were normalized by treating maximal concentration of agonist as 100%. Data were plotted and analyzed using nonlinear regression in GraphPad Prism software.

#### Calcium assay

In order to assess the Gαq-coupling and activation, we performed calcium assay using Fluo4-NW dye (Invitrogen, Cat. no. F36206). HEK293 cells were transfected with FLAG-tagged receptor encoded in the pcDNA3.1 construct (3.5 μg). After 24 h of transfection, cells were seeded at a density of 125,000 cells per 50 μl in each well of a 96-well plate. Following seeding, the plate was incubated at 37°C and 5% CO_2_ for 1 h to allow the cells to settle down. After 1 h, the plate was removed from the incubator and 50 μLof freshly prepared 2X dye loading solution was subsequently added to each well. The plate was again incubated at 37°C and 5% CO_2_ for an additional 30 min followed by 30 min incubation at room temperature. The fluorescence was recorded using multimode plate reader (EnSpire; Perkin Elmer) at excitation wavelength of 494 nm and emission wavelength of 516 nm. The human bradykinin receptor (B_2_R) was used as a positive control in the experiment. Data were normalized by subtracting values of fluorescence recorded after ligand treatment with values of baseline fluorescence and time kinetics showing calcium response was plotted in the GraphPad Prism software.

#### Chemical cross-linking and co-immunoprecipitation

For measuring agonist dependent βarr recruitment by respective receptor constructs, chemical crosslinking was performed following previously published protocol (Ghosh et al., 2019). Briefly, HEK293 cells were transfected with FLAG-tagged receptor. 48 h post-transfection, cells were serum starved in DMEM for at least 6 h followed by stimulation with 100 nM of C5a for C5aR2 and CCL7 for D6R. Cells were lysed in a homogenizer in lysis buffer (20 mM HEPES, pH 7.4,100 mM NaCl, Protease and Phosphatase inhibitor cocktail). Purified βarr1 or βarr2 (2.5 μg) was added to the lysate and allowed to incubate for 1 h at room temperature. Freshly prepared amine reactive crosslinker, DSP (Sigma, Cat. no. D3669) at a final concentration of 1.5 mM was added to the reaction mixture and incubated for an additional 45 min at room temperature to allow cross-linking of receptor-βarr complex. Following incubation with DSP, the reaction was quenched using 1 M Tris, pH 8.0. 1% (v/v) MNG (maltose neopentyl glycol) was added for solubilisation of receptor-βarr complex at room temperature for 1 h. In order to capture the complex, pre-equilibrated FLAG M1 antibody beads were added and incubated for additional 2 h at 4°C. Beads were thoroughly washed to remove any non-specific binding and the receptor-βarr complex were finally eluted in FLAG-EDTA solution (20 mM HEPES, 150 mM NaCl, 2 mM EDTA, 0.01% MNG, 250 μg ml^-1^ FLAG-peptide) and further incubated for 20 min at room temperature. Receptor and βarr were probed by immunoblotting by using rabbit mAb anti-βarr antibody (1:5000, CST, Cat. no. 4674). The blot was stripped and then reprobed for FLAG-tagged receptor using anti-FLAG antibody (1:2000, Sigma, Cat. no. A8592). For co-IP with overexpressed βarr1/2, 3.5 μg of either βarr1 or βarr2 was coexpressed along with the receptor in HEK293 cells followed by cross-linking, IP and visualization as described above. Data were quantified using ImageLab software (Bio-Rad) and were analyzed using appropriate statistical analysis in GraphPad prism.

#### Confocal microscopy

In order to visualize βarr recruitment and trafficking for D6R and C5aR2, confocal microscopy was used following the protocol described previously (Dwivedi-Agnihotri et al., 2020). Briefly, HEK293 cells were transfected with receptor (3.5 μg), βarr1-mYFP or βarr2-mYFP (3.5 μg). For D6R mutants the constructs were normalized for surface expression and HEK293 cells were transfected with D6R^WT^ (200 ng), D6R^1-351^ (5 μg) and D6R^1-342^ (7 μg). After 24 h, cells were seeded at 1 million density on to 0.01% poly-D-lysine (Sigma, Cat. no. P0899) pre-treated confocal dishes (SPL Lifesciences, Cat. no. 100350). Cells were allowed to attach to the plate for 24 h, prior to agonist stimulation cells were serum starved for at least 6 h. For fixed cell imaging, cells were fixed after starvation with 4% PFA (Sigma, Cat. no. P6148) in 1X phosphate-buffered saline (PBS, Sigma, Cat. no. D1283) for 20 min at 4°C. Fixed cells were thoroughly washed with 1XPBS and then the cells were incubated in 3% BSA with 0.1% Triton X-100 in 1XPBS (Sigma, Cat. no. 9002-93-1) for permeabilization. For staining FLAG tagged receptors we used anti-FLAG M1 antibody (1:100) labeled with DyLight594 (Thermo Scientific, Cat. no. 46412) dye in presence of 2 mM CaCl_2_. Cells were thoroughly washed with 1XPBS having 2 mM CaCl_2_. Finally, for nuclear staining, DAPI stain (5 μg ml^-1^ (Sigma, Cat. no. D9542)) was used for 10 min at room temperature. After extensive washing, cells on coverslips were mounted on slides with VectaShield HardSet mounting medium (VectaShield, Cat. no. H-1400). Confocal imaging of all samples was done using Zeiss LSM 710 NLO confocal microscope where samples were housed on a motorized XY stage with a CO_2_ enclosure and a temperature-controlled platform equipped with 32x array GaAsP descanned detector (Zeiss). A Ti: sapphire laser (Coherent) was used for exciting the DAPI channel, a Multi-Line argon laser source is used for the green channel (mYFP), and for the red channel (DyLight 594), a diode pump solid state laser source was used. All microscopic setting including laser intensity and pinhole opening were kept in the same range for a parallel set of experiments. For avoiding any spectral overlap between two channels filter excitation regions and bandwidths were adjusted accordingly. Images were scanned in line scan mode and acquired images were processed post imaging in ZEN lite (ZEN-blue/ZEN-black) software suite from ZEISS. For quantifying receptor co-localization with βarrs, the Pearson’s correlation coefficient was measured using JACoP plugin in ImageJ suite. Three regions of interest per cell were analyzed for each receptor at membrane and endosomes both and the mean ± SEM of PCCs are mentioned for respective receptors in the figure legends along with the number of cells and the number of independent experiments. For quantifying βarr trafficking confocal images captured in 1 to 8 min and 9 to 30 min after agonist stimulation were categorized into early and late time points, respectively. The scoring of βarr localization was done on the basis of mYFP fluorescence either in the plasma membrane (surface localized) or in the punctate structures in the cytoplasm (internalized). In cells where βarrs were seen in both, the membrane and in punctate structures, cells having more than three punctae in the cytoplasm were scored under internalized category. All the experiments were repeated at least three times independently on different days, and the data are plotted as percentage of βarr localization from more than 500 cells for each condition. To avoid any sort of bias in manual counting, the same set of images was analyzed by three different individuals in a blinded fashion and cross-checked. All data were plotted in GraphPad Prism software.

#### Isolation of C5aR-βarr1-Fab30 complex and negative staining electron microscopy

N-terminal Flag-tagged C5aR2 was expressed in cultured *Sf9* cells together with GRK6 and βarr1 using the baculovirus expression system. 66 h post-infection, cells were stimulated with C5a followed by stabilization of the complex using Fab30. Subsequently, the complex was purified using anti-Flag M1 antibody agarose and size exclusion chromatography as described previously for the analogous β_2_AR-βarr1 complex (Shukla et al., 2014). Prior to staining, the C5aR2-βarr1-Fab30 protein complex was diluted to 0.04 mg ml^-1^ in buffer containing 20 mM HEPES, pH7.4 and 150 mM NaCl. Negative staining was performed in accordance with previously published protocols (Peisley and Skiniotis, 2015). In brief, 3.5 μL of the protein complex was applied onto glow discharged formvar/carbon coated 300 mesh copper grids (PELCO, Ted Pella) and blotted off after adsorption of the sample for 1 min using a filter paper. Staining was done with freshly prepared 0.75% (w/v) uranyl formate stain for 45 s. The negatively stained samples were imaged with a FEI Tecnai G2 12 Twin TEM (LaB6, 120 kV) equipped with a Gatan CCD camera (4k x 4k) at 30,000x magnification. Approximately, 10,000 particles were picked manually from 253 micrographs using e2boxer.py within the EMAN2.31 software suite (Tang et al., 2007). 2D classification of the picked particles was performed with ISAC2 (Yang et al., 2012) within the SPHIRE suite (Moriya et al., 2017) using the box files generated from EMAN2.31.

#### NanoBiT-β-arrestin recruitment assay

The effect of GRKs on agonist-induced βarr activation was measured by a NanoBiT-βarr recruitment assay. Specifically, the parental HEK293A, GRK2/3-KO, GRK5/6 and GRK2/3/5/6-KO cells (Arveseth et al., 2021) were seeded and transfection was performed by following the same procedures as described in “NanoBiT-based G protein dissociation assay” section. For the βarr recruitment assay, 100 ng (hereafter per well in a 6-well plate) of an N-terminally LgBiT-fused βarr1/2 plasmid and 100 ng (C5aR1; with 400 ng of an empty vector) or 500 ng (C5aR2, CCR2 and D6R) of a test GPCR plasmid with the N-terminal HA-derived signal sequence and FLAG-epitope tag and a C-terminal SmBiT (ssHA-FLAG-GPCR-SmBiT). The transfected cells were subjected to the NanoBiT luminescent measurement as described above. Luminescence counts from 10 min to 15 min after compound addition were used for the calculation.

#### Detection of D6R basal phosphorylation by pIMAGO assay

To detect agonist independent basal phosphorylation in D6R, pIMAGO phosphoprotein detection kit from Sigma (Cat. no. 18419) was used and receptor phosphorylation was detected as per the manufacturer’s protocol. Briefly, HEK293 cells were transfected with 7 μg D6 receptor DNAcomplexed with 21 μg PEI. 48 h after transfection, cells were serum-starved for 6 h followed by stimulation with 200 nM CCL7 for 30 min and harvested in 1XPBS. Post stimulation, cells were lysed in buffer containing 50 mM HEPES (pH 7.4), 150 mM NaCl, 10% glycerol (v/v), 1% NP40, 2 mM EDTA, 1X phosSTOP and 1X protease inhibitor cocktail (Roche, Cat. no. 04693116001) for 2h at room temperature. The lysate was cleared by centrifugation and transferred to a separate tube already containing pre-equilibrated M1-FLAG beads supplemented with 2 mM CaCl_2_. The receptor was enriched by performing FLAG-immunoprecipitation as described previously. Afterward, the protein was eluted in FLAG elution buffer containing 20 mM HEPES pH 7.5, 150 mM NaCl, 2 mM EDTA, 0.06% NP40 and 250 μg ml^-1^ FLAG peptide. Subsequently, protein loading dye was added to each sample, followed by the addition of 5X IAA solution to a 1X final concentration from the pIMAGO kit. The samples were incubated at room temperature for 15 min in dark. Eluted samples were subjected to SDS-PAGE followed by western blotting. The membrane was blocked in 1X blocking buffer for 1 h followed by incubation with pIMAGO reagent (1:1000, prepared in 1X pIMAGO buffer) for 1 h. The membrane was washed thrice with 1X wash buffer and once with 1XTBST (5 min each wash). The PVDF membrane was incubated with avidin-HRP (1:1000, prepared in 1X blocking buffer) for 1h at room temperature and washed thrice with 1XTBST (5 min each wash). The signal was detected using Promega ECL solution on chemidoc (BioRad). Blot was stripped and re-probed for total receptor using HRP conjugated anti-FLAG M2-antibody (Sigma, 1:5000). The signal was normalized with respect to total receptor and quantified using ImageLab software (BioRad).

To assess the role of D6R C terminus in basal receptor phosphorylation, the receptor was truncated at C terminus at two positions (i.e., 1-342 and 1-351) by inserting a STOP codon by site-directed mutagenesis (NEB, Cat. no. E0554). The surface expression of WT and truncated receptor constructs were normalized to similar levels by DNA titration in HEK293 cells. Relative surface expression of all the constructs was measured by whole cell-based surface ELISA as described previously. For the detection of basal phosphorylation in D6R-WT and mutants, 50%–60% confluent HEK293 cells were transfected with D6R-WT (200 ng) and mutant receptor DNA complexed with 21 μg PEI (1-342: 7 μg, 1-351: 5 μg). For each construct, 5x10 cm HEK293 plates were transfected. 48 h post-transfection, cells were harvested in 1XPBS and lysed in NP40-lysis buffer. Receptor phosphorylation was detected using a western blot based pIMAGO- phosphoprotein detection kit as mentioned in the previous section.

To identify the specific determinants of βarr interaction in D6RC terminus, 50%–60% confluent HEK293 cells were transfected with either D6R-WT (200 ng) or mutants (1-342: 5 μg and 1-351: 5 μg) and βarr2 (2 μg). The surface expression of WT and mutant receptor constructs was normalized to similar levels as mentioned in the previous section. 48h post-transfection, cells were serum-starved for 6 h followed by stimulation with 100 nM CCL7 for 30 min. Post-stimulation, cells were harvested in 1XPBS and proceeded for chemical crosslinking. Cells were lysed by Dounce homogenization in 20 mM HEPES pH 7.5, 350 mM NaCl, 1XPhosSTOP, and 1X complete protease inhibitor cocktail). This was followed by the addition of freshly prepared dithiobis(succinimidyl-propionate) to a final concentration of 1.5 mM. Cell lysates were tumbled at room temperature for 40 min and the reaction was quenched by 1 M Tris pH 8.5. Afterward, lysates were solubilized in 1% MNG (w/v) at room temperature for 1.5 h and centrifuged at 15000 rpm for 10 min. Cleared lysates were supplemented with CaCl_2_ to a final concentration of 2 mM followed by the addition of pre-equilibrated M1-FLAG beads to the lysate. The samples were tumbled at room temperature for 1.5 h to allow bead binding and beads were washed 3 times each with low salt buffer (20 mM HEPES pH7.5,150 mM NaCl, 2 mM CaCl_2_, and 0.01% MNG) and high salt buffer (20 mM HEPES pH7.5, 350 mM NaCl, 2 mM CaCl_2_ and 0.01% MNG) alternately. The bound proteins were eluted in FLAG-elution buffer containing 20mM HEPES pH 7.5, 150mM NaCl, 2mM EDTA, 0.01% MNG and 250 μg ml^-1^ FLAG peptide. Eluted βarr2 was detected by western blotting using rabbit anti-βarr mAb (1:5000, CST, Cat. no. 4674). The blots were stripped and re-probed for receptor with HRP-coupled anti-FLAG M2 antibody (1:5000). The blots were developed on Chemidoc (Bio-Rad) and quantified using ImageLab software (Bio-Rad).

#### Ib30 NanoBiT Assay

We measured ligand-induced βarr conformational change recognized by Intrabody 30 (Ib30) using NanoBiT assay (Baidya et al., 2020a). Ib30 and βarr1 were N-terminally fused to LgBiT and SmBiT respectively with the 15-amino acid flexible linker and inserted into the pCAGGS plasmid. The receptor pair C5aR1 and C5aR2 exhibited matched cell surface expression at DNA concentration of 0.25 μg and 3 μg respectively. Similarly, cells transfected with 0.5 μg DNA of D6R and 3 μg CCR2 showed comparable surface expression. For NanoBiT assay, HEK293 cells at a density of 3 million were transfected with receptor (DNA concentration as mentioned above), LgBiT-Ib30 (5 μg) and SmBiT βarr1 (2 μg) using PEI (Polyethylenimine; 1 mg ml^-1^) as transfection agent at DNA:PEI ratio of 1:3. After 16-18 h of transfection, cells were harvested in PBS solution containing 0.5 mM EDTA and centrifuged. Cells were resuspended in 3 mL assay buffer (HBSS buffer with 0.01% BSA and 5 mM HEPES, pH 7.4) containing 10 μM coelenterazine (Goldbio, Cat. no: CZ05) at final concentration. The cells were then seeded in a white, clear-bottom, 96 well plate at a density of 0.7 X 10^5^ cells per100 μL per well. The plate was kept at 37°C for 90 min in the CO_2_ incubator followed by incubation at room temperature for 30min. Basal reading was read on luminescence mode of multi-plate reader (Victor X4). The cells were then stimulated with varying doses of each ligand (C5a and CCL7) ranging from 0.1 pM to 1 μM (6x stock, 20 μl per well) prepared in drug buffer (HBSS buffer with 5 mM HEPES, pH 7.4). Luminescence was recorded for 60 min immediately after addition of ligand. The initial counts of 4-10 cycles were averaged and basal corrected. Fold increase was calculated with respect to vehicle control (unstimulated values) and analyzed using nonlinear regression four-parameter sigmoidal concentration-response curve in GraphPad Prism software.

#### FlAsH BRET experiments

HEK293SL cells were cultured in DMEM supplemented with 10% FBS and 20 μg ml^-1^ gentamicin, and grown at 37°C in 5% CO_2_ and 90% humidity. Cells were seeded at a density of 1.5 × 10^5^ cells per well in 6-well plate and were transiently transfected the next day with C5aR1, C5aR2, D6R, or CCR2 and βarr2-FlAsH constructs using conventional calcium phosphate co-precipitation method. One day post-transfection, cells were detached and seeded in poly-ornithine-coated white 96-well plates at a density of 2.5 × 10^4^ cells per well in media. The next day, cells were washed and incubated for 1 h with Tyrode’s buffer (140 mM NaCl, 2.7 mM KCl, 1 mM CaCl_2_, 12 mM NaHCO_3_, 5.6 mM D-glucose, 0.5 mM MgCl_2_, 0.37 mM NaH_2_PO_4_, 25 mM HEPES, pH 7.4) at room temperature. FlAsH labeling was performed as previously described (Lee et al., 2016). Briefly, 1.75 μL of FlAsH-EDT_2_ stock reagent was mixed with 3.5 μL of 25 mM EDT solution in DMSO and left for 10 min at room temperature. 100 μL of Tyrode’s buffer was then added and left for 5 min at room temperature. The volume was then adjusted to 5 mL with Tyrode’s buffer to complete the labeling solution. Cells were washed with Tyrode’s buffer and incubated with 60 μL of labeling solution per well for 1 h at 37°C. Cells were then washed twice with BAL wash buffer followed by another wash with Tyrode’s buffer. Next, 90 μL of Tyrode’s buffer was added per well and incubated for 1 h at 37°C. Cells were stimulated with 1 μM C5a or CCL7 ligand for 10 min, with six consecutive BRET measurements taken every minute after 5 min stimulation. Cell-permeable substrate coelenterazine H (final concentration of 2 μM) was added 3 min prior to BRET measurements, with triplicates for each condition. BRET measurements were performed using a Victor X (PerkinElmer) plate reader with a filter set (center wavelength/band width) of 460/25 nm (donor) and 535/25 nm (acceptor). BRET ratios were determined by dividing the intensity of light emitted by the acceptor over the intensity of light emitted by the donor. The net BRET ratio is calculated by subtracting the background BRET ratio (unlabeled) from the FlAsH-EDT_2_-labeled BRET ratio. The Δnet BRET is then obtained by dividing the stimulated net BRET ratio by the vehicle net BRET ratio.

#### ERK1/2 phosphorylation assay in HEK293 cells

Agonist-induced ERK1/2 MAP kinase phosphorylation was carried out following the protocol described previously (Kumari et al., 2019). Briefly, HEK293 cells expressing the indicated receptors were seeded into a 6-well plate at a density of 1 million cells per well. Cells were serum-starved for 12 h followed by stimulation with the indicated concentration of corresponding ligands at specific time points. To study the effect of pertussis toxin (PTX) on basal ERK phosphorylation of C5aR2, cells were treated with 100 ng ml^-1^ PTX (in starvation media) for 12 h prior to ligand stimulation. Similarly, to study the effect of MEK-inhibitor (U0126) on basal ERK phosphorylation of C5aR2, cells were pretreated with 10 μM U0126 for 30 min before ligand stimulation. After the completion of the time course, the media was aspirated, and cells were lysed in 100 μl 2x SDS dye per well. Cell lysates were heated at 95°C for 15 min followed by centrifugation at 15000 rpm for 10 min. 10 μL of lysate was loaded per well and separated in SDS-PAGE followed by western blotting. Blots were blocked in 5% BSA (in TBST) for 1 h and incubated overnight with rabbit phospho-ERK (CST, Cat. no. 9101) primary antibody at 1:5000 dilution. Blots were washed thrice with TBST for 10 min each and incubated with anti-rabbit HRP-coupled secondary antibody (1:10000, Genscript), Cat. No. A00098 for 1 h. Blots were washed again with TBST for three times and developed with Promega ECL solution (Cat. no. W1015) on chemidoc (BioRad). Blots were stripped with low pH stripping buffer and then re-probed for total ERK using rabbit total ERK (CST, Cat. no. 9102) primary antibody at 1:5000 dilution.

#### Phospho-antibody array

A phospho-antibody array (Full Moon Biosystems) consisting of 1318 antibodies against proteins from multiple signaling pathways were used to discover potential signaling pathways downstream of receptors investigated here. The samples were prepared as per the manufacturer’s instruction and sent to Full Moon Biosystems for further analysis. Briefly, HEK293 cells stably expressing the receptor was stimulated with saturating concentration of ligands (C5a, 100 nM for C5aR1 and C5aR2; CCL7, 100 nM for D6R) for 10 min and then harvested using 1 mL of ice-cold 1X PBS supplemented with 0.01% Phosphatase inhibitor (PhosSTOP, Roche, Cat. no. 04906845001). Pellets corresponding to 10 plates of a 10 cm plate were pooled together and centrifuged at 5,000 rpm for 5 min at 4°C. The supernatant was discarded and the pellets were washed again with 1 mL of cold 1X PBS to remove any traces of media. HEK293 cells stably expressing the receptor under non-stimulation conditions were used as a control. Three independent set of pellets comprising of cells pooled from the unstimulated conditions and stimulated conditions were prepared following similar conditions and sent to Full Moon Biosystems for phosphoarray and analysis. The antibody array was done using a kit (Cat. no. KAS02). Briefly cells was lysed and centrifuged to obtain a clear lysate. Prior labeling of the proteins in the lysate with biotin, buffer was exchanged for ensuring proper biotinylation. The amount of total protein was analyzed using BCA estimation for both unstimulated and stimulated conditions. Subsequently, equal amount of biotinylated proteins were allowed to bind with the immobilized antibodies coated on a glass slide. After rigorous washing, Cy3-streptavidin was used to detect the bound proteins to respective antibodies. The antibody array slide was finally detected using a microarray scanner. Fold increase in signal was obtained after dividing the fluorescence signal emitted from respective antibody spots for stimulated sample by corresponding signal from unstimulated sample. A list of all the target proteins, their phosphorylation sites being detected using this array, and the fold change over basal (agonist-treatment versus no-treatment) are presented in Table S1.

#### MS-based Phosphoproteomics

##### Preparation of cell lysate

For MS-based phosphoproteomics of D6R, HEK293 cells stably expressing the D6R were grown at a confluency of ~70%. Cells were serum starved for at least 6 h prior to stimulation. Cells were then stimulated for 10 min with 100 nM of CCL7. Media was then aspirated and cells were washed with 1XTBS containing 0.01% of phosphatase inhibitors. Cells corresponding to 10 plates each were scraped and collected and pelleted in a 15 mL falcon. Three independent sets of unstimulated and stimulated cell pellets each were prepared. The pellets were then treated with 6 M Gn-HCL/0.1 M Tris (pH 8.5) plus phosphatase inhibitors and resuspended well. The lysate was then boiled at 95°C for 10 min, followed by sonication for breaking the nucleic acids and reducing the viscosity of slimy material. After sonication, lysate was again boiled at 95°Cfor 5 min and then spun at 15,000 rpm for 20 min at room temperature. The supernatant was collected carefully leaving behind the insoluble debris. Protein concentration was estimated by BCA using the same lysis buffer as a blank solution. Lysates corresponding to 5 mg each bioreplicate was sent to V-Proteomics for Mass Spectrometry analysis.

##### Sample preparation and phosphopeptide enrichment

For sample preparation, 5mg of the Gn-HCL protein lysate were first reduced with 5 mM TCEP and further alkylated with 50 mM iodoacetamide. Alkylated proteins were further diluted using 50mM Ammonium Bicarbonate to bring final Gn-HCL concentration to 0.6M and then digested with trypsin (1:50, trypsin: lysate ratio, Promega) for 16 h at 37°C. The overnight digests were clarified with brief spin and the supernatant pH was adjusted around pH2 using 10% TFA. Sep-Pak (Waters) columns were washed with methanol and washing buffer (2%acetonitrile/0.1%TFA) followed by loading of total peptides. The column was washed 3 times with washing buffer (2%acetonitrile/0.1%TFA) and final peptide elution was performed using high acetonitrile containing Elution buffers (50% and 80%acetonitrile/0.1%TFA). The peptide mixture was dried using speed vac. The dried peptide pellet was dissolved using Phthalic acid Buffer (0.1% P.Acid/20% Water/80% Acetonitrile, 2.5%TFA) and mixed with TiO2 (Titansphere 5 μm, GL sciences) beads and mixed on a rotator for two hours. The beads were washed two times with Phthalic acid Buffer followed by washing with 80% acetonitrile/0.1%TFA and finally 0.1% TFA. Phosphopeptides were eluted using 0.3M NH_4_OH and the pH was adjusted around 2 using 50% TFA. The Phosphopeptides were dried using speed vac and further clarified using C18 mini columns as per above mentioned buffers.

##### Mass-spectrometry (MS) analysis and data acquisition

The purified phosphopeptide dried pellet was finally resuspended in Buffer-A (5% acetonitrile / 0.1% formic acid). For the mass spectrometry analysis of peptide mixtures, all the experiments were performed using EASY-nLC1200 system (Thermo Fisher Scientific) coupled to QExactive mass spectrometer (Thermo Fisher Scientific) equipped with nanoelectrospray ion source. 1 μg of the phosphopeptide mixture was loaded on 2cm pre-column (Acclaim Pepmap c18, 3 micron resin) and resolved using Easyspray column (2 micron resin, 25 cm length). The peptides were loaded with Buffer A and eluted with a 0%–40% gradient of Buffer-B (95% acetonitrile/0.1% Formic acid) at a flow rate of 300 nL min^-1^ for 105 min. The QExactive was operated using the Top10 HCD data-dependent acquisition mode with a full scan resolution of 70,000 at m/z 400. MS/MS scans were acquired at a resolution of 17500 at m/z 400. Lock mass option was enabled for polydimethylcyclosiloxane (PCM) ions (m/z = 445.120025) for internal recalibration during the run. MS data was acquired using a data-dependent top10 method dynamically choosing the most abundant precursor ions from the survey scan.

##### MS data processing and statistical analysis

For data analysis, all six raw files (3 sets of stimulated and 3 sets of unstimulated samples) were analyzed with Proteome Discoverer 2.2 against the Uniprot Human reference proteome database (containing 20162 entries). For Sequest HT and MS Amanda 2.0 search, the precursor and fragment mass tolerances were set at 10 and 20 ppm respectively. The protease used to generate peptides, i.e., enzyme specificity was set for trypsin/P (cleavage at the C terminus of “K/R: unless followed by “P”) along with maximum missed cleavages value of two. Carbamidomethyl on cysteine as fixed modification and oxidation of methionine, N-terminal acetylation and phosphorylation on Serine, Threonine and Tyrosine were considered as variable modifications for database search. Both peptide spectrum match and protein false discovery rate were set to 0.01 FDR and determined using percolator node. Relative protein quantification of the proteins was performed using Minora feature detector node of Proteome Discoverer 2.2 with default settings and considering only high PSM (peptide spectrum matches) confidence. Based on uniprot accession number Pfam, KEGG pathways and GO annotations were assigned for the list of identified proteins. Also, for high sensitivity-phospho site localization to be detected for individual site, ptmRS node was considered. Among all the proteins detected phosphoproteins were selected and all the respective phosphopeptides were further analyzed based on their relative label free quantification (LFQ) values. The data matrix was imported in perseus software (version 1.6.0.7) and data was further filtered for those phosphopeptides where LFQ values were available in at least 4 samples among the total 6 samples (3 bioreplicates of both stimulated and unstimulated samples). The LFQ abundance values of these filtered peptides were log2 transformed and imputation was applied using perseus default settings (width 0.3, downshift 1.8) where missing values were replaced by random numbers that are drawn from a normal distribution. Student’s t test was applied between stimulated and unstimulated group samples using a p value significance threshold level of 0.05 and the test results were represented as volcano plot. Z-score normalization was applied using median abundance values and Student’s t test significant phospho-peptide abundance values were used for hierarchical clustering of rows and/or columns (Distance: Euclidean, Linkage: average, Preprocess: k-means, Number of clusters:300, Maximum iterations:10) in order to generate heatmap/Clustergram.

##### Comparison of D6R data with previously published studies

In order to get a functional insight from the robust data generated from phospho-proteomics and phospho-array, we also analyzed additional datasets from the literature namely the phospho-proteomics dataset for CCR2, a chemokine receptor (Huang et al., 2020a), and a βarr biased phospho-proteomics dataset carried out on the AT1aR stimulated with a biased agonist SII (Christensen et al., 2010; Xiao et al., 2010). The rationale behind including the above datasets is because D6R is a chemokine receptor with exclusive bias toward βarr. Therefore, comparing the above-mentioned dataset can bring out some common features of βarr signaling involving chemokine receptors. Moreover, the selected hits from D6R not matching with these previous datasets should allow us to identify additional βarr-mediated signaling outcomes arising from atypical chemokine receptors. After comparing all the datasets with multifind utility in mightymacros excel, overlapping hits were identified. Furthermore, a list of proteins common to both, phospho-antibody array and phosphoproteomics, was generated with their phospho-sites labeled, it was submitted to kinase enrichment analysis tool KEA 2.0 (https://www.maayanlab.net/KEA2/). The significantly enriched hits were listed based on their P values. The listed proteins were further analyzed in STRING database to identify protein-protein interaction to understand the pathways involved. Using this criterion, we selected and screened multiple antibodies from list for immunoblotting based validation from cell lysate of which three proteins i.e., protein kinase D1 (PKD1/PRKD1), cofilin, and the platelet derived growth factor receptor β (PGGFR-β) were validated.

#### Validation of D6R phospho-protein hits

For validation of phospho-array and phospho-proteomics hits, stable cell lines expressing D6R were seeded at 3 million per 10 cm plate. Cells were serum-starved with 20 mM HEPES (pH 7.4) and 1% BSA in serum-free DMEM media for 16-18h. Cells were then stimulated with 200 nM CCL7 for indicated time points. Cells were lysed in buffer containing 50 mM HEPES (pH 7.4), 150 mM NaCl, 10% glycerol (v/v), 1% NP40, 2 mM EDTA, 1X phosSTOP and 1X protease inhibitor cocktail for 1h at room temperature. The lysate was cleared by centrifugation and solubilized proteins were estimated with BCA method (G Bioscience). Approximately 90-100 μg of each sample was loaded on 4%-20% precast gradient gel (Bio-Rad) and resolved proteins were transferred to a PVDF membrane. After blocking with 3% BSA in 1X TBST blots were incubated with primary antibody (phospho-Cofilin^S3^, 1:1000; phospho-PKD^S744/748^1:1000, phospho-PDGFR^Y751^, 1:1000). Blots were developed in ChemiDocMP Gel Imaging System (Bio-Rad) after incubating the blots in anti-rabbit HRP antibody (1:5000) for 1h at room temperature.

To evaluate the role of βarr isoforms in phosphorylation of these hits, D6R or C5aR2 plasmids were transfected in control-, βarr1- and βarr2-shRNA expressing cell lines at 7 μg. Serum-starvation, stimulation, and sample preparation were performed as mentioned previously. About 90-100 μg of cell lysates were run on 4%–20% precast gradient gel and western blotting was performed as per the previous protocol. PVDF membranes were probed for phospho-Cofilin^S3^ (CST, Cat. no. 3313, 1:1000), phospho-PKD^S744/748^ (CST, Cat. no. 2054, 1:1000), phospho-PDGFR^Y751^(CST, Cat. no. 4549, 1:1000), phospho-P90RSK^S380^ (CST, Cat. no. 11989, 1:500). Blots were stripped with low pH stripping buffer and then re-probed for total RSK using RSK1/RSK2/RSK3 rabbit monoclonal primary antibody at 1:2500 dilution (CST, Cat. no. 9355S,) or for β-actin (Sigma, Cat. no. A3854, 1:50000). Phospho-site specific signal was normalized with respect to the total RSK or β-actin signal.

#### Ligand-induced p90RSK phosphorylation in HEK293 cells

In order to measure C5a-induced p90RSK phosphorylation, HEK293 cells stably expressing C5aR2 were seeded in 10 cm culture dishes at a density of 5 million. After 24 h, cells were subjected to serum starvation for 16 h followed by stimulation with 100 nM C5a for indicated time points and harvested in PBS. Subsequently, cells were lysed in 200 μL of 2XSDS reducing buffer, and lysates were heated at 95°C for 30 min followed by centrifugation at 15000 rpm for 15 min. Afterward, 10 μL of cell lysate was loaded in each well and separated on SDS-PAGE followed by western blotting. The PVDF membranes were blocked in 5% BSA (in TBST) for 1 h followed by overnight incubation with phosphorylation site-specific p90RSK primary antibodies (phospho-Thr^359^, Cat. no. 8753, 1:500; phospho-Thr^573^, Cat. no. 9346S, 1:500; phospho-SerS^380^, CST, Cat. no. 11989, 1:500). Next day, blots were washed thrice with TBST for 10min each and incubated with anti-rabbit HRP-coupled secondary antibody (Genscript, Cat. no. A00098, 1:2000) for 1 h. The secondary antibody was rinsed off by washing the blots again with TBST for three times and developed with Promega ECL solution on chemidoc (BioRad). Blots were stripped with low pH stripping buffer and then re-probed for total RSK using RSK1/RSK2/RSK3 rabbit monoclonal primary antibody at 1:2500 dilution (CST, Cat. no. 9355S). Phospho-site specific signal was normalized with respect to the total RSK signal.

#### Human monocyte-derived macrophages

Human monocyte-derived macrophages (HMDM) were derived and cultured following the previously described protocol (Li et al., 2020a, 2020b). Briefly, human buffy coat blood from anonymous healthy donors was obtained through the Australian Red Cross Blood Service (Brisbane, Australia). Human CD14^+^ monocytes were isolated from blood using Lymphoprep density centrifugation (STEMCELL, Melbourne, Australia) followed by CD14^+^ MACS separation (Miltenyi Biotec, Sydney, Australia). The isolated monocytes were cultured in Iscove’s Modified Dulbecco’s Medium (IMDM) containing 10% FBS, 100 U ml^-1^ penicillin, 100 μg ml^-1^ streptomycin and 15 ng ml^-1^ recombinant human macrophage colony stimulating factor (Lonza, Melbourne, Australia) on 100 mm square dishes (Bio-strategy, Brisbane, Australia). The adherent differentiated HMDMs were harvested by gentle scraping on Day 6-7.

#### In-cell western assays on HMDMs

In-cell western assays were performed following the technical guidelines provided by LI-COR Biosciences (Lincoln, USA). Briefly, HMDMs were seeded (80,000 per well) in poly D-lysine-coated (Merck, Perth, Australia) black-wall clear-bottom tissue culture 96-well plates (Corning, Corning, USA) for 24 h and serum-starved overnight. All ligands were prepared in serum-free IMDM containing 0.1% BSA (Merck, Perth, Australia). Cells were first pre-treated with the C5aR1 antagonist PMX53 (10 μM)for20min (37°C, 5% CO_2_) before stimulation with recombinant human C5a (Sino Biological, Beijing, China) or P32 (100 μM) for 10 min at room temperature. The media was removed and the cells were fixed using 4% paraformaldehyde (Alfa Aesar, Haverhill, USA) (10min, room temperature). Upon gentle washing with DPBS, the cells were permeabilised using ice-cold methanol (10 min, room temperature) and then blocked using Odyssey Blocking Buffer in TBS (LI-COR Biosciences) (1.5h, room temperature). The cells were then stained with the indicated primary antibodies at 4°C overnight (phospho-p90RSK^S380^, CST, Cat. no. 11989S, 1:800; phospho-p90RSK^T359^, CST, Cat. no. 8753S, 1:200; phospho-p90RSK^T573^, CST, Cat. no. 9346S, 1:200; Human/Mouse/Rat RSK Pan Specific Antibody, R&D Systems, Cat. no. RDSMAB2056, 1:200; phospho-p^44/42^ MAPK-ERK1/2^T202/Y204^, CST, Cat. no. 9101S, 1:250). Upon further washing with DPBS containing 0.1% Tween-20, the cells were stained with IRDye 680RD donkey anti-rabbit secondary antibody (Cat. no. 926-68073, 1:1000,) and/or IRDye 800CW donkey anti-mouse IgG secondary antibody (Cat. no. 926-32212, 1:1000,) (LI-COR Biosciences, Lincoln, USA) for 1.5h at room temperature. The plate was then washed with DPBS containing 0.1% Tween-20 and blotted dry. For fluorescence quantification, the plate was read on a Tecan Spark 20M microplate reader (Ex/Em: 667 nm/707 nm for IRDye 680RD and 770 nm/810 nm for IRDye 800CW, respectively) (Tecan, Männedorf, Switzerland).

#### PMN mobilization assay

Wild-type (WT), C5aR1−/− and C5aR2−/− mice on a C57BL/6J genetic background (n = 5-15) were administered with recombinant mouse C5a (Sino Biological, China) at a dose of 50 μg kg-1 via intravenous injection (tail vein). After C5a injection, one drop of blood was collected from the tail tip to make a blood smear on a slide at 0, 15, 30 and 60 min. Blood smears were stained using a Microscopy Hemacolor® Rapid Staining of Blood Smear Kit (Merck, Germany). Briefly, blood smears were fixed in Hemacolor® Solution 1 (methanol). The slides were then stained with Hemacolor® Solution 2 (Eosin Y), followed by Hemacolor® Solution 3 (Azur B). The slides were washed with 1 x PBS (pH 7.2) and mounted with dibutylphthalate polystyrene xylene. Using a 20x/0.4 NA objective on an Olympus CX21 microscope, first 400 white blood cells were counted for each slide, and the proportion of PMNs (i.e., cells containing granules that are light violet) was then calculated as previously described (Wu et al., 2020).

### Quantification and Statistical Analysis

All the experiments were conducted at least three times and data (mean ± SEM) were plotted and analyzed using GraphPad Prism software (Prism 8.0). Heatmaps were plotted in Python 3.7 using appropriate libraries. The data were normalized with respect to proper experimental controls and appropriate statistical analyses were performed. The details of normalization, replicates, and statistical analysis are mentioned in the corresponding figure legends.

## Supplementary Material

Supplemental information can be found online at https://doi.org/10.1016/j.molcel.2021.09.007.

FigureS1-S7

## Data Availability

The original raw data for immunoblots and confocal micrographs have been deposited in Mendeley Data (https://doi.org/10.17632/nzpd6k32gz.1). Phosphoproteomics data are deposited in the ProteomeXchange repository (PXD027887) and has been made publicly available as of the date of publication. This paper does not report any original code. Any additional information required to reanalyze the data reported in this paper is available from the lead contact upon reasonable request.

## Highlights

- D6R and C5aR2 couple to and signal through β-arrestins but not G proteins
- D6R and C5aR2 exhibit distinct contribution of GRKs for β-arrestin recruitment
- D6R and C5aR2 induce distinct β-arrestin conformations compared to GPCR counterparts
- D6R and C5aR2 offer a novel experimental framework to study biased agonism

## In brief

Pandey et al. discover that D6R and C5aR2 couple to β-arrestins but not G-proteins and thus represent natural examples of β-arrestin-biased 7TMRs. These receptors also exhibit distinct preferences for GRK engagement and β-arrestin conformations compared to prototypical GPCR counterparts and provide a novel framework to study biased agonism.
