# Supplementary material for: Intrinsic bias at non-canonical, β-arrestin-coupled seven transmembrane receptors
Source: Mol Cell. Author manuscript; Available in PMC 2022 Jun 4. (PMC7612807; doi:10.1016/j.molcel.2021.09.007)
Supplement: FigureS1-S7 [file EMS145237-supplement-FigureS1_S7.pdf]

Molecular Cell, Volume *81*

Supplemental information

Intrinsic bias at non-canonical,

β-arrestin-coupled

seven transmembrane receptors

Shubhi Pandey, Punita Kumari, Mithu Baidya, Ryoji Kise, Yubo Cao, Hemlata Dwivedi-Agnihotri, Ramanuj Banerjee, Xaria X. Li, Cedric S. Cui, John D. Lee, Kouki Kawakami, Jagannath Maharana, Ashutosh Ranjan, Madhu Chaturvedi, Gagan Deep Jhingan, Stéphane A. Laporte, Trent M. Woodruff, Asuka Inoue, and Arun K. Shukla

Figure S1

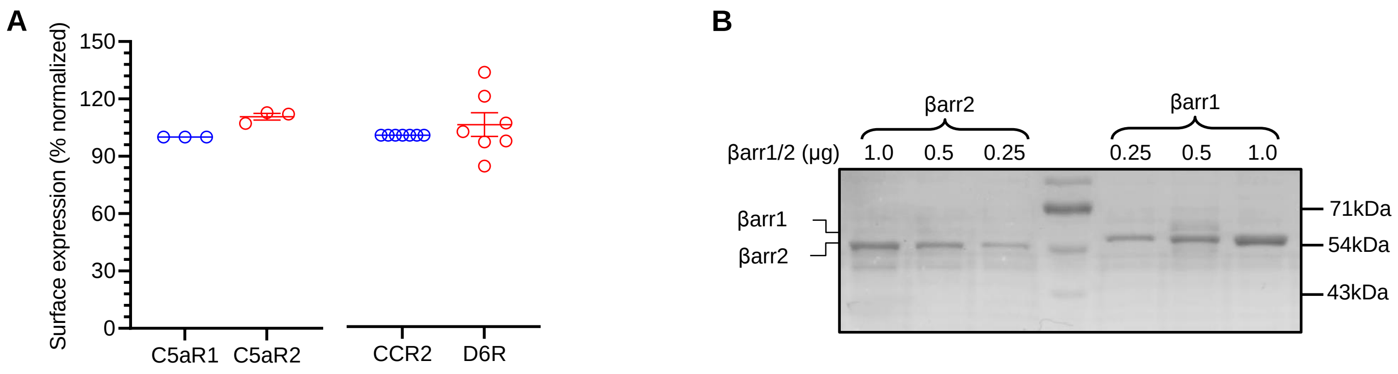

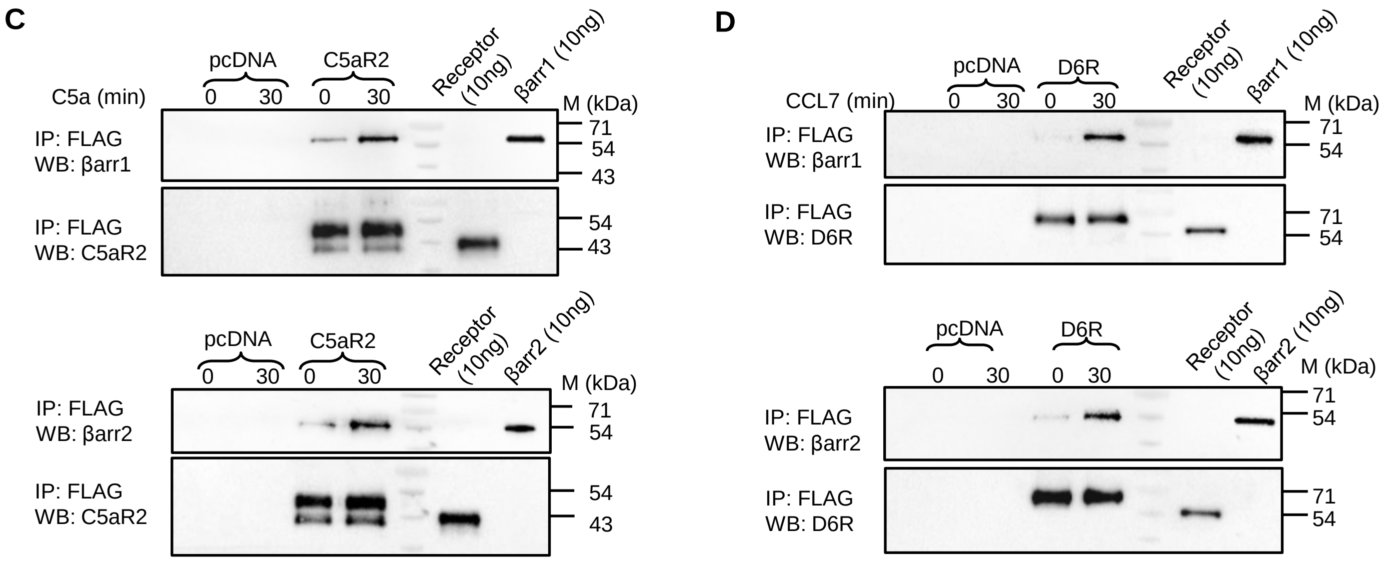

	βarr1 (ng) 5.9 ± 0.79	βarr2 (ng) 12.6 ± 0.65
C5aR2	35.8 ± 3.85	35.3 ± 3.47
Molar ratio	~ 0.2 ± 0.04	~ 0.4 ± 0.05

	βarr1(ng) 7.9 ± 0.64	βarr2 (ng) 15.2 ± 3.42
D6R (ng)	94.9 ± 35.56	50.0 ± 6.94
Molar ratio	~ 0.1 ± 0.05	~0.3 ± 0.11

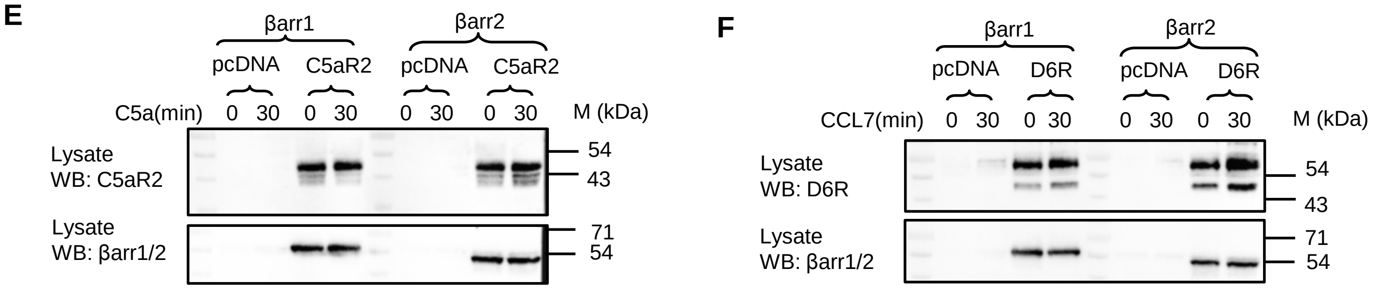

Figure S1, related to Figure 1 and 2. Surface expression of receptors and interaction with βarrs.

A. Surface expression of the indicated receptors used in G-protein dissociation assay as measured by flow-cytometry using an anti-FLAG primary antibody and Alexa Fluor-488-coupled secondary antibody. Values of mean fluorescence intensity (MFI) from approximately 20,000 cells per sample were used for analysis.

B. Normalization of purified βarr1/2 used in co-IP experiments. Different amounts of purified βarr1 and 2 used in the co-IP experiments were separated by SDS-PAGE and visualized using Coomassie staining.

C-D. Interaction of βarrs with C5aR2 and D6R measured by co-IP assay. Lysate from HEK-293 cells expressing the indicated receptors were mixed with purified βarr1/2 (2.5μg) followed by cross-linking and co-IP using anti-Flag M1 antibody agarose. Receptors and βarrs were visualized by Western blot using the corresponding antibodies, and quantified by densitometry. Indicated amounts of purified receptor (C5aR2) and βarr1/2 were also included on the blot to quantify the pull-down of βarrs and receptors in co-IP experiments. The numbers presented in tabular format indicate densitometry-based quantification of βarrs and receptor pull-down in the co-IP assay. Immunoblot images presented in Figure 2A are taken from the same blots that are presented here.

E-F. Expression level of C5aR2 and D6R in the cellular lysate, and equal amounts of βarr1/2 used in co-IP experiments, were confirmed by Western blotting using anti-Flag M2 antibody and anti-βarr antibody.

Figure S2

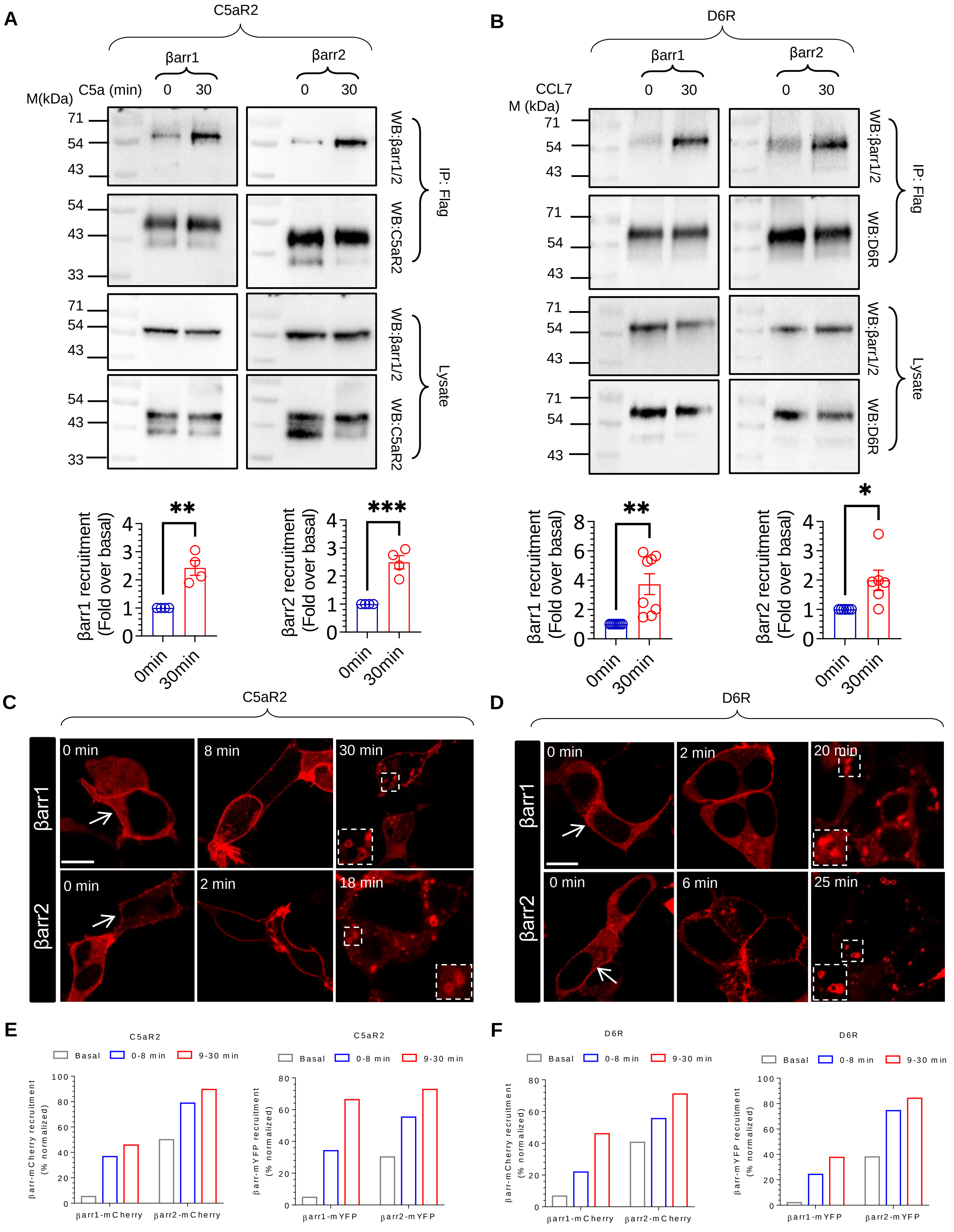
A
C5aR2
βarr1
βarr2
M(kDa)
C5a (min)
0
30
0
30
71
54
43
54
43
33
71
54
43
54
43
33
WB:βarr1/2
WB:C5aR2
WB:βarr1/2
WB:C5aR2
IP: Flag
Lysate
B
D6R
βarr1
βarr2
CCL7
M (kDa)
0
30
0
30
71
54
43
71
54
43
71
54
43
71
54
43
WB:βarr1/2
WB:D6R
WB:βarr1/2
WB:D6R
IP: Flag
Lysate
βarr1 recruitment (Fold over basal)
βarr2 recruitment (Fold over basal)
**
***
**
*
0min
30min
C
C5aR2
βarr1
βarr2
0 min
8 min
30 min
0 min
2 min
18 min
D
D6R
βarr1
βarr2
0 min
2 min
20 min
0 min
6 min
25 min
E
C5aR2
Basal
0-8 min
9-30 min
βarr-mCherry recruitment (% normalized)
βarr1-mCherry
βarr2-mCherry
βarr-mYFP recruitment (% normalized)
βarr1-mYFP
βarr2-mYFP
F
D6R

Figure S2, related to Figure 2. Interaction and trafficking of βarrs upon C5aR2 and D6R stimulation.

A-B. Interaction of βarrs with C5aR2 and D6R upon co-expression measured using co-IP assay. Lysate from HEK-293 cells expressing the indicated receptors and βarrs were subjected to cross-linking and co-IP using anti-Flag M1 antibody agarose. Receptors and βarrs were visualized by Western blot using the corresponding antibodies, and quantified by densitometry. The bar graph represent densitometry-based quantification of 4-8 independent experiments analyzed using t-test (*p<0.05; **p<0.01; ***p<0.001).

C-D. Agonist-induced trafficking of mCherry-tagged βarr1/2 for C5aR2 and D6R. HEK-293 cells expressing indicated receptors along with mCherry-tagged βarr1 or βarr2 were observed for βarr trafficking in live cells upon specific agonist stimulation (D6R: CCL7 100nM; C5aR2: C5a 100nM). Both D6R and C5aR2 show constitutive βarr recruitment at the membrane under basal condition but trafficking to endosomes occurs only on ligand stimulation. There is apparent preference for βarr2 isoform over βarr1 in both D6R and C5aR2. Micrographs are representative images from three independent experiments at indicated time-points (scale bar, 10μm).

E-F. Quantification of agonist-induced βarr1/2 translocation measured by confocal microscopy. Agonist-induced localization of both mCherry- and mYFP-tagged βarr1 and 2 in D6R and C5aR2 were measured by confocal microscopy as described in material and methods section. βarr localization at the cell surface and in punctate structures (endosomal vesicles) was scored under basal and agonist-induced conditions at indicated time-points as a readout of βarr recruitment. Confocal images were classified in three groups i.e. 0min, 1-8min and 9-30min post-agonist stimulation to reflect basal, early and late time-frames, respectively. Approximately five hundred cells from three independent experiments were scored for each condition, and the graphs represent % of cells showing βarr recruitment (i.e. localization at the cell surface, in punctate structures, or both).

Figure S3

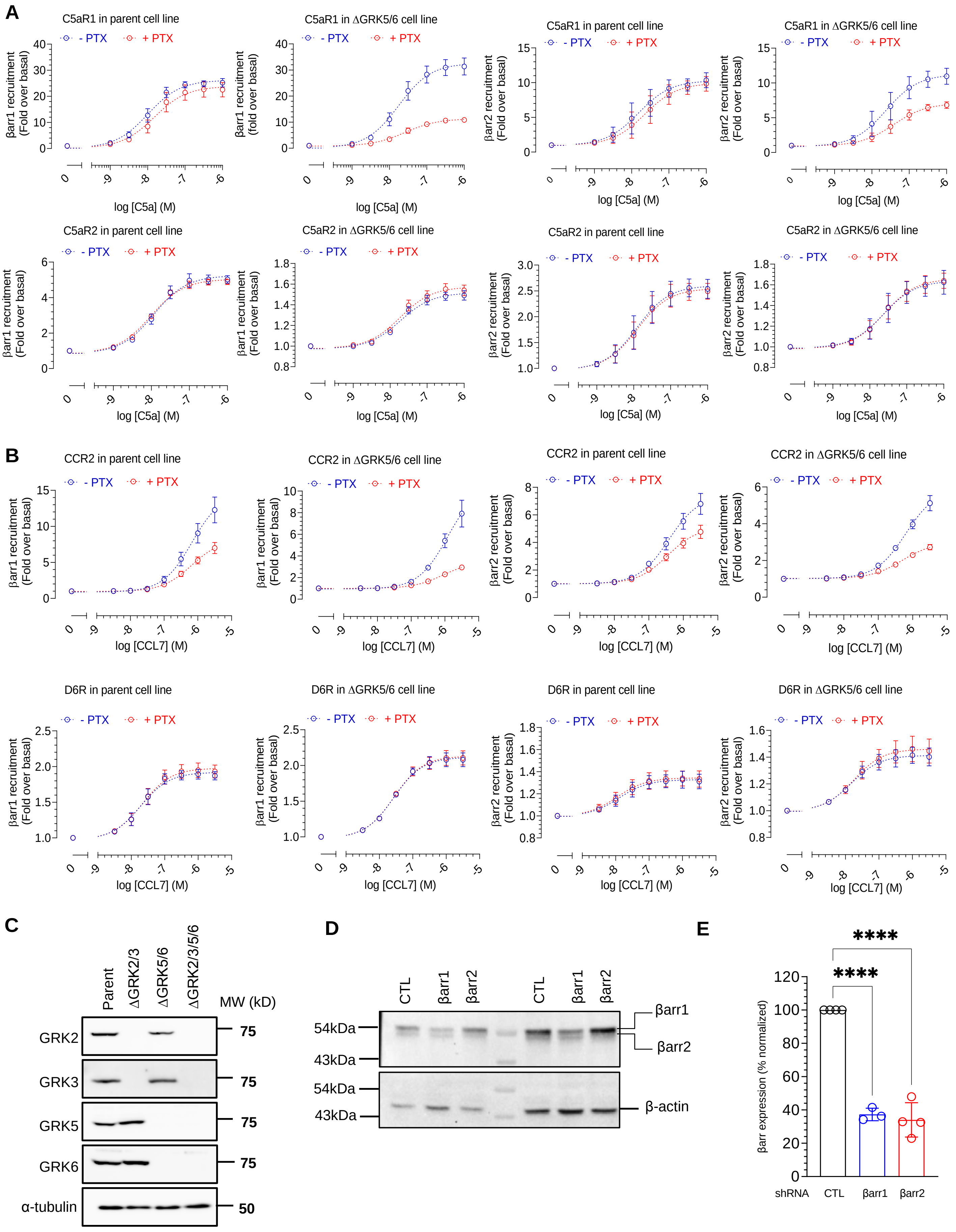
A
C5aR1 in parent cell line
C5aR1 in ΔGRK5/6 cell line
C5aR2 in parent cell line
C5aR2 in ΔGRK5/6 cell line
- PTX
+ PTX
βarr1 recruitment (Fold over basal)
βarr2 recruitment (Fold over basal)
log [C5a] (M)
B
CCR2 in parent cell line
CCR2 in ΔGRK5/6 cell line
D6R in parent cell line
D6R in ΔGRK5/6 cell line
log [CCL7] (M)
C
Parent
ΔGRK2/3
ΔGRK5/6
ΔGRK2/3/5/6
MW (kD)
GRK2
GRK3
GRK5
GRK6
α-tubulin
75
50
D
CTL
βarr1
βarr2
54kDa
43kDa
β-actin
E
βarr expression (% normalized)
shRNA
****

Figure S3, related to Figure 3, 5 and 7. βarr recruitment and efficiency of βarr/GRK depletion

A-B. The interaction of βarrs with the indicated receptors in either parental or ΔGRK5/6 cell line was measured using the NanoBiT assay in presence or absence of PTX. Data (mean ± SEM) from three independent experiments normalized with respect to vehicle-treatment are presented here.

C. CRISPR-Cas9-mediated knock-out of GRKs as measured by Western blot using the corresponding antibodies and α-tubulin was used as loading control.

D. Efficiency of βarr knock-down in shRNA transfected stable HEK-293 cells as measured by Western blot using anti-βarr antibody. β-actin expression is used as loading control.

E. Densitometry-based quantification of Western blot data from 3-4 independent experiments, normalized with shRNA-CTL (control) condition suggests 60-70% knock-down efficiency.

Figure S4

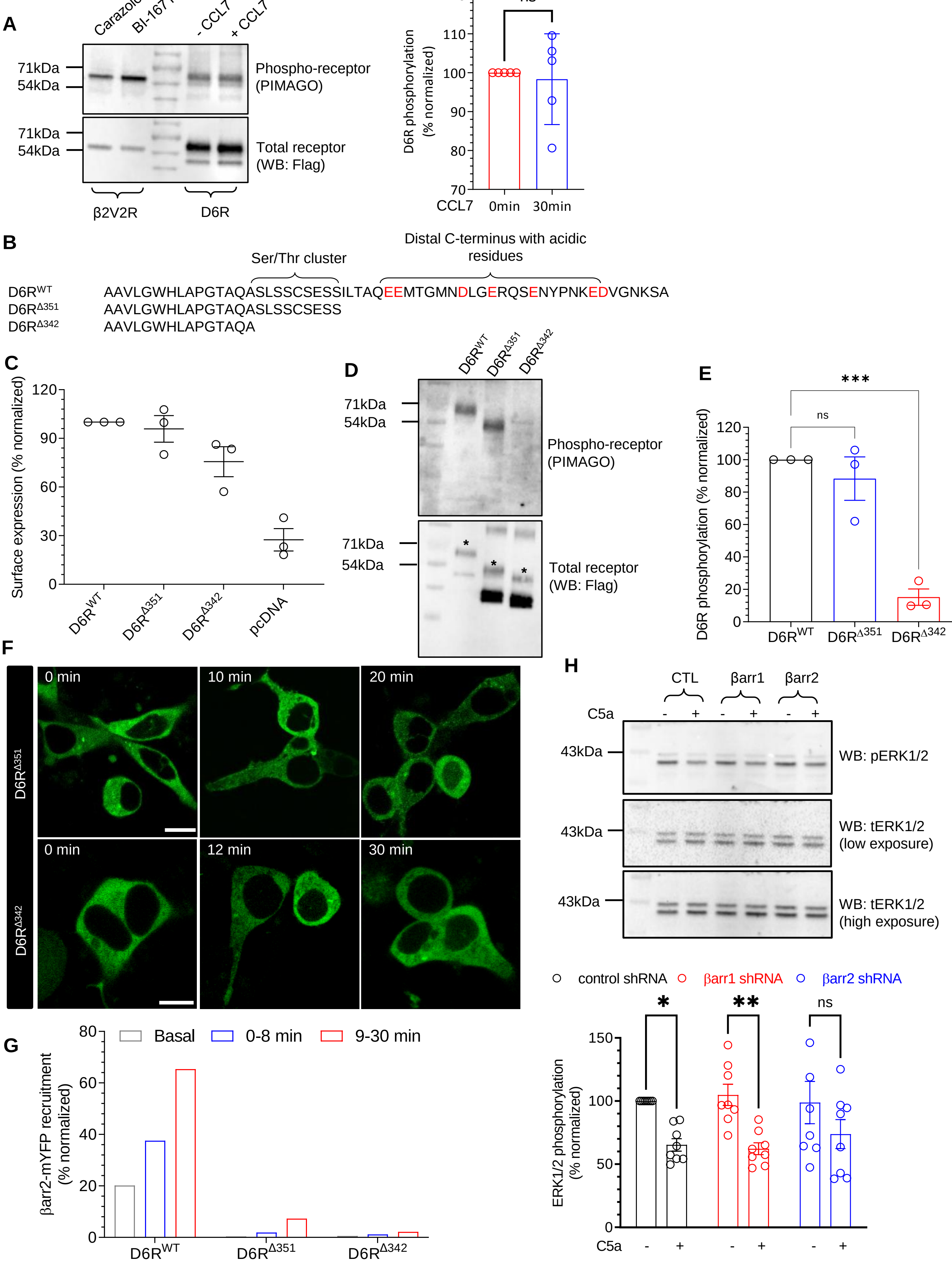
A
Carazolol
BI-167107
- CCL7
+ CCL7
71kDa
54kDa
Phospho-receptor (PIMAGO)
Total receptor (WB: Flag)
β2V2R
D6R
ns
D6R phosphorylation (% normalized)
CCL7
0min
30min
B
Ser/Thr cluster
Distal C-terminus with acidic residues
D6R^WT AAVLGWHLAPGTAQASLSSCSESSILTAQEEMTGMNDLGERQSENYPNKEDVGNKSA
D6R^Δ351 AAVLGWHLAPGTAQASLSSCSESS
D6R^Δ342 AAVLGWHLAPGTAQA
C
Surface expression (% normalized)
D6R^WT
D6R^Δ351
D6R^Δ342
pcDNA
D
Phospho-receptor (PIMAGO)
Total receptor (WB: Flag)
E
***
ns
D6R phosphorylation (% normalized)
F
0 min
10 min
20 min
12 min
30 min
G
Basal
0-8 min
9-30 min
βarr2-mYFP recruitment (% normalized)
H
CTL
βarr1
βarr2
C5a
43kDa
WB: pERK1/2
WB: tERK1/2 (low exposure)
WB: tERK1/2 (high exposure)
control shRNA
βarr1 shRNA
βarr2 shRNA
*
**
ERK1/2 phosphorylation (% normalized)

Figure S4, related to Figure 3. and 5. D6R phosphorylation, βarr recruitment and C5aR2-mediated ERK1/2 phosphorylation.

A. D6R is constitutively phosphorylated as measured using PIMAGO kit, and its phosphorylation does not change upon ligand (CCL7) stimulation (n=5). As a control, a chimeric β2-adrenergic receptor construct was used, which exhibits agonist-induced phosphorylation**.** A part of this data is presented as Figure 3C.

B. Carboxyl-terminus truncation of D6R lacking acidic residues and Ser/Thr clutser.

C. Surface expression of D6R constructs expressed in HEK-293 as measured by whole cell ELISA. Data (mean±SEM) from three independent measurements, normalized with wild-type D6R (treated as 100%) are presented here.

D-E. The primary phosphorylation determinants of D6R are localized in the region between Ser^{351} and Ser^{342} as the receptor truncation at Ser^{342} but not at Ser^{351} abolishes constitutive phosphorylation. All three constructs were expression-matched with respect to surface expression in HEK-293 cells, and only the matured receptor population (indicated with an asterisk on the anti-Flag blot) was used for normalization ($D6R^{WT}$ as 100%, n=3, One-Way Anova, ***$p<0.001$).

F. HEK-293 cells expressing the indicated receptor constructs and βarr2-mYFP were stimulated with agonist (100nM CCL7) and localization of βarr2 was monitored by confocal microscopy (Scale bar is 10μm).

G. For assessing βarr recruitment and trafficking in D6R deletion mutants, βarr2 localization was manually scored in HEK293 cells from multiple fields from at least three independent experiments. Confocal images captured are grouped in three sections i.e. 0min, 1-8min and 9-30min post-agonist stimulation to reflect basal, early and late time-frames, respectively. The bar graph indicates the % of cells showing βarr recruitment (i.e. localization at the cell surface, in punctate structures or both).

H. Effect of βarr depletion on C5aR2-mediated ERK1/2 phosphorylation. C5aR2-expressing cells exhibit an elevated level of pERK1/2, which is reduced upon C5a-stimulation. shRNA-mediated depletion of βarr2 but not βarr1 attenuates the C5a-induced lowering of pERK1/2 while knock-down of either βarrs have no significant effect on basal pERK1/2. The upper panel shows a representative image from eight independent experiments and the lower panel show densitometry-based quantification from eight independent experiments normalized with respect to the basal pERK1/2 under control shRNA conditions (One-Way ANOVA, *$p<0.05$; **$p<0.01$).

Figure S5

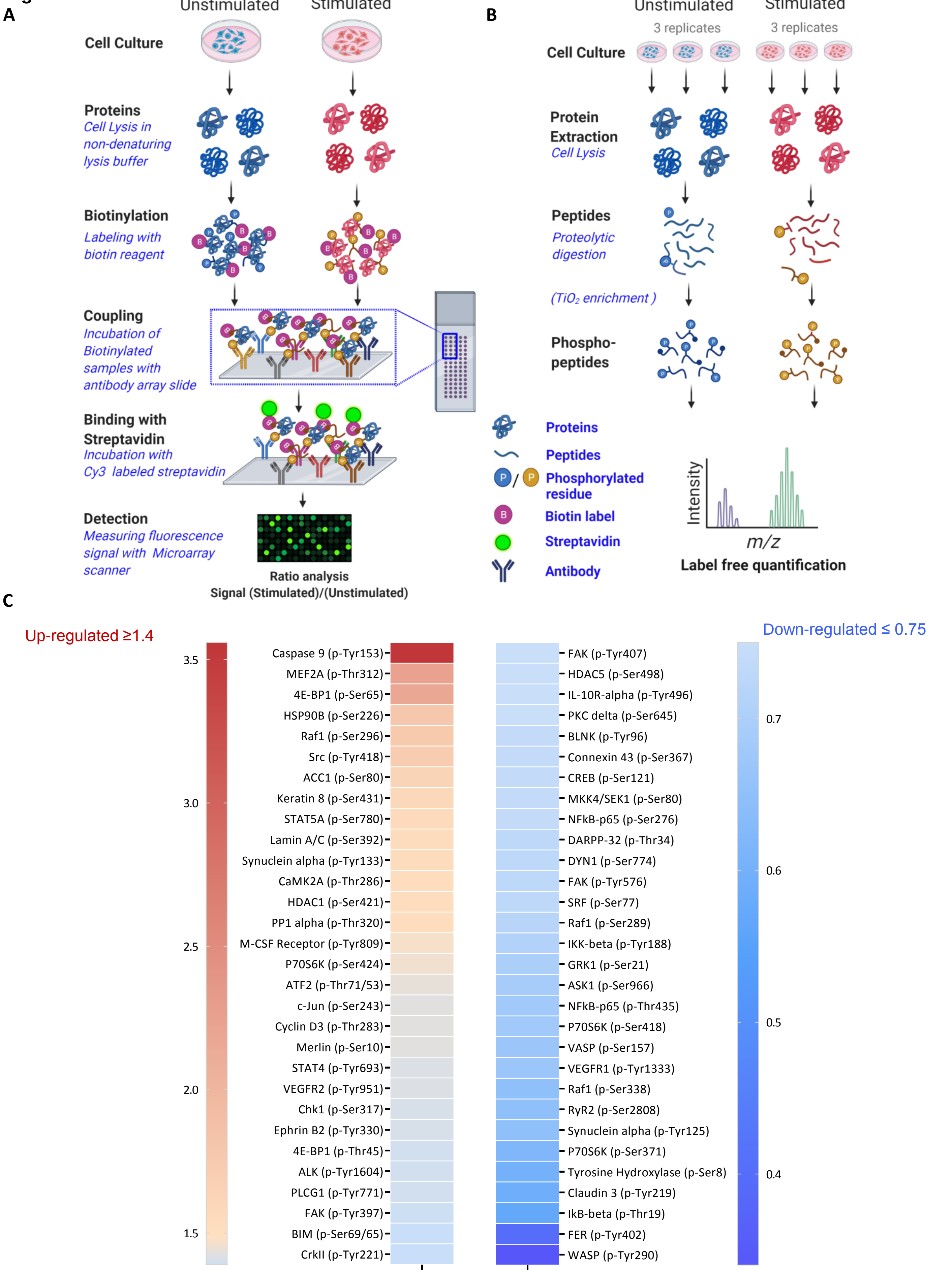
A
Unstimulated
Stimulated
Cell Culture
Proteins
Cell Lysis in non-denaturing lysis buffer
Biotinylation
Labeling with biotin reagent
Coupling
Incubation of Biotinylated samples with antibody array slide
Binding with Streptavidin
Incubation with Cy3 labeled streptavidin
Detection
Measuring fluorescence signal with Microarray scanner
Ratio analysis
Signal (Stimulated)/(Unstimulated)
B
Unstimulated
3 replicates
Stimulated
3 replicates
Cell Culture
Protein Extraction
Cell Lysis
Peptides
Proteolytic digestion
(TiO_2 enrichment)
Phospho-peptides
Proteins
Peptides
Phosphorylated residue
Biotin label
Streptavidin
Antibody
Intensity
m/z
Label free quantification
C
Up-regulated ≥1.4
3.5
3.0
2.5
2.0
1.5
Caspase 9 (p-Tyr153)
MEF2A (p-Thr312)
4E-BP1 (p-Ser65)
HSP90B (p-Ser226)
Raf1 (p-Ser296)
Src (p-Tyr418)
ACC1 (p-Ser80)
Keratin 8 (p-Ser431)
STAT5A (p-Ser780)
Lamin A/C (p-Ser392)
Synuclein alpha (p-Tyr133)
CaMK2A (p-Thr286)
HDAC1 (p-Ser421)
PP1 alpha (p-Thr320)
M-CSF Receptor (p-Tyr809)
P70S6K (p-Ser424)
ATF2 (p-Thr71/53)
c-Jun (p-Ser243)
Cyclin D3 (p-Thr283)
Merlin (p-Ser10)
STAT4 (p-Tyr693)
VEGFR2 (p-Tyr951)
Chk1 (p-Ser317)
Ephrin B2 (p-Tyr330)
4E-BP1 (p-Thr45)
ALK (p-Tyr1604)
PLCG1 (p-Tyr771)
FAK (p-Tyr397)
BIM (p-Ser69/65)
CrkII (p-Tyr221)
Down-regulated ≤ 0.75
FAK (p-Tyr407)
HDAC5 (p-Ser498)
IL-10R-alpha (p-Tyr496)
PKC delta (p-Ser645)
BLNK (p-Tyr96)
Connexin 43 (p-Ser367)
CREB (p-Ser121)
MKK4/SEK1 (p-Ser80)
NFkB-p65 (p-Ser276)
DARPP-32 (p-Thr34)
DYN1 (p-Ser774)
FAK (p-Tyr576)
SRF (p-Ser77)
Raf1 (p-Ser289)
IKK-beta (p-Tyr188)
GRK1 (p-Ser21)
ASK1 (p-Ser966)
NFkB-p65 (p-Thr435)
P70S6K (p-Ser418)
VASP (p-Ser157)
VEGFR1 (p-Tyr1333)
Raf1 (p-Ser338)
RyR2 (p-Ser2808)
Synuclein alpha (p-Tyr125)
P70S6K (p-Ser371)
Tyrosine Hydroxylase (p-Ser8)
Claudin 3 (p-Tyr219)
IkB-beta (p-Thr19)
FER (p-Tyr402)
WASP (p-Tyr290)
0.7
0.6
0.5
0.4

Figure S5, related to Figure 6 and 7. Phospho-antibody array and phosphoproteomics experiments.

A-B. Schematic representation of phospho-antibody array and phosphoproteomics experiments. Samples prepared from HEK-293 cells expressing D6R under basal and agonist-stimulated conditions were processed for phospho-antibody array and MS-based phospho-proteomics following the steps outlined in these schematics. Further experimental and technical details are provided in the corresponding Materials and Methods section.

C. The summary of Phospho-antibody array hits identified for D6R. Cellular lysates prepared from D6R-expressing cells with and without CCL7-stimulation (100nM, 10min) were used for the array. The change in phosphorylation status of cellular proteins (ratio between basal and agonist-treatment of ≤0.75 for down-regulated hits and ≥1.4 for upregulated hits) is plotted in GraphPad. The phosphorylation sites for each of the indicated proteins used in this array are indicated in parenthesis.

Figure S6

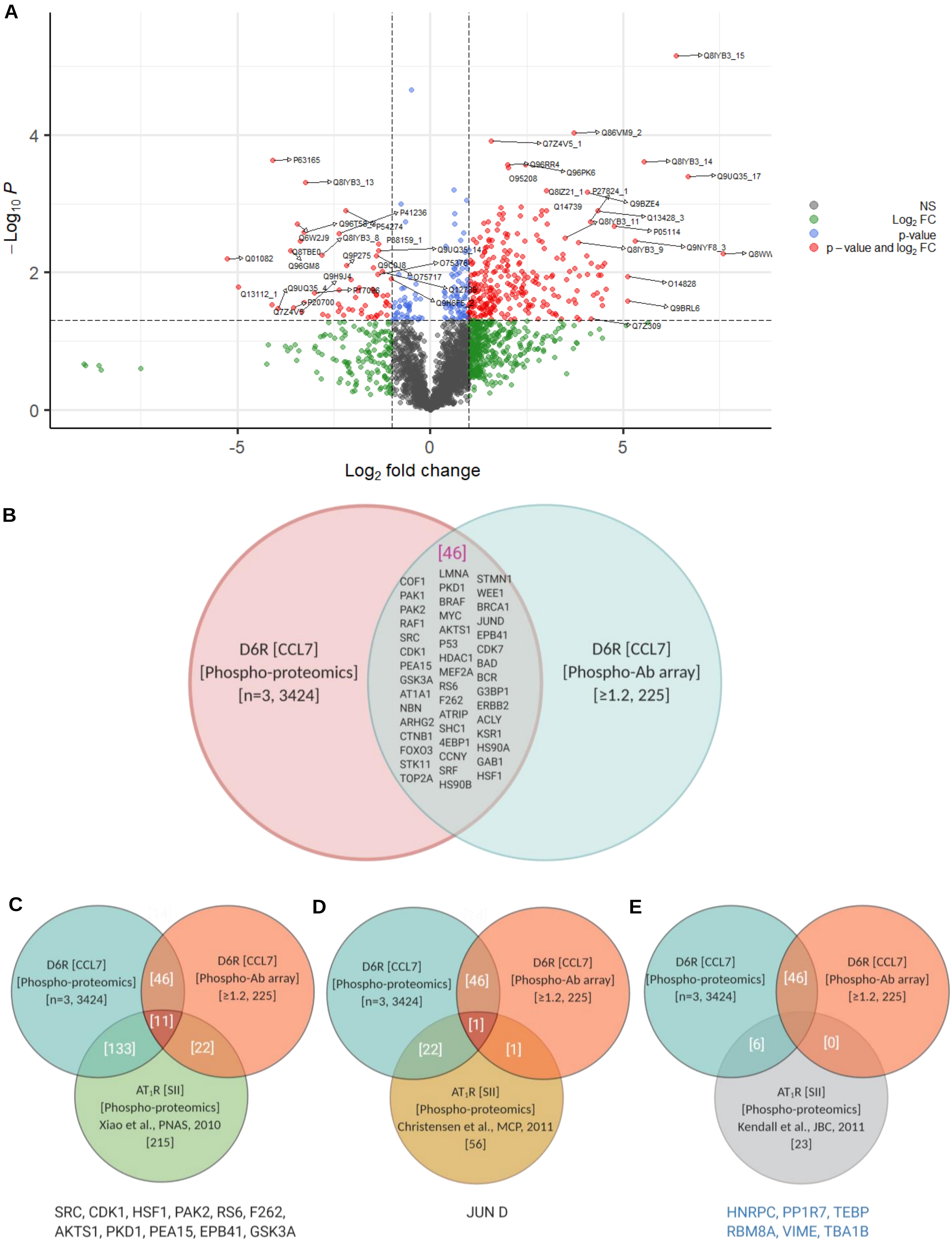

A
$-\log_{10} P$
Log2 fold change
NS
Log2 FC
p-value
p – value and log2 FC
Q8IYB3_15
Q86VM9_2
Q7Z4V5_1
P63165
Q8IYB3_14
Q96RR4
Q96PK6
Q9UQ35_17
Q8IYB3_13
O95208
Q8IZ21_1
P27824_1
Q9BZE4
Q14739
Q13428_3
Q8IYB3_11
P05114
Q8IYB3_9
Q9NYF8_3
P41236
Q96T58
P54274
Q6W2J9
Q8IYB3_8
P68159_1
Q9UQ35_14
Q8TBE0
Q01082
Q96GM8
Q9P275
O75376
Q9C0J8
O75717
Q9H9J4
O14828
Q13112_1
Q9UQ35_4
P17096
Q12789
Q9H6F5
P20700
Q7Z4V5
Q9BRL6
Q7Z309
B
D6R [CCL7]
[Phospho-proteomics]
[n=3, 3424]
[46]
COF1
PAK1
PAK2
RAF1
SRC
CDK1
PEA15
GSK3A
AT1A1
NBN
ARHG2
CTNB1
FOXO3
STK11
TOP2A
LMNA
PKD1
BRAF
MYC
AKTS1
P53
HDAC1
MEF2A
RS6
F262
ATRIP
SHC1
4EBP1
CCNY
SRF
HS90B
STMN1
WEE1
BRCA1
JUND
EPB41
CDK7
BAD
BCR
G3BP1
ERBB2
ACLY
KSR1
HS90A
GAB1
HSF1
D6R [CCL7]
[Phospho-Ab array]
[≥1.2, 225]
C
D6R [CCL7]
[Phospho-proteomics]
[n=3, 3424]
[46]
D6R [CCL7]
[Phospho-Ab array]
[≥1.2, 225]
[11]
[133]
[22]
AT1R [SII]
[Phospho-proteomics]
Xiao et al., PNAS, 2010
[215]
SRC, CDK1, HSF1, PAK2, RS6, F262,
AKTS1, PKD1, PEA15, EPB41, GSK3A
D
D6R [CCL7]
[Phospho-proteomics]
[n=3, 3424]
[46]
D6R [CCL7]
[Phospho-Ab array]
[≥1.2, 225]
[1]
[22]
[1]
AT1R [SII]
[Phospho-proteomics]
Christensen et al., MCP, 2011
[56]
JUN D
E
D6R [CCL7]
[Phospho-proteomics]
[n=3, 3424]
[46]
D6R [CCL7]
[Phospho-Ab array]
[≥1.2, 225]
[6]
[0]
AT1R [SII]
[Phospho-proteomics]
Kendall et al., JBC, 2011
[23]
HNRPC, PP1R7, TEBP
RBM8A, VIME, TBA1B

Figure S6, related to Figure 6. Summary and analysis of the phosphoproteomics data on D6R.

A. Volcano plot generated based on D6R phosphoproteomics data. The Plot was generated by inference based t-test statistics (p-value significance threshold level of 0.05). The estimated fold changes (x-axis) versus the -log 10 p-values (y-axis) for each phosphopeptide are indicated on the plot. NS, non-significant; red dots demonstrate t-test significant proteins.

B-E. Analysis of phospho-antibody array and phosphoproteomics hits. The panel B shows a Venn diagram comparing the protein identified in the phosphoproteomics and phospho-antibody array screens performed on D6R expressing cells. The common proteins are indicated in the overlapping region with the generic gene symbols. Panels C-E show the Venn diagram comparing the D6R phospho-hits with previous studies carried out on AT1R stimulated with a βarr-biased ligand, SII. The common hits identified in this analysis are listed and indicated by their gene symbol. COF1, Cofilin-1; PAK1, p21-activated kinase 1; PAK2, p21-activated kinase 2; RAF1, Proto-oncogene c-RAF; SRC, Tyrosine-protein kinase Src; CDK1, Cyclin-dependent kinase 1; PEA15, Astrocytic phosphoprotein PEA-15; GSK3A, Glycogen synthase kinase-3 alpha; AT1A1, Sodium/potassium-transporting ATPase subunit alpha-1; NBN, Nibrin; ARHG2, Rho guanine nucleotide exchange factor 2; CTNB1, Catenin beta-1; FOXO3, Forkhead box protein O3; STK11, Serine/threonine kinase 11; TOP2A, DNA topoisomerase 2-alpha; LMNA, Prelamin-A/C; PKD1 (PRKD1), Protein kinase D1; BRAF, proto-oncogene B-Raf; MYC, Myc proto-oncogene protein; AKTS1, Proline-rich AKT1 substrate 1; p53, Tumor protein P53; HDAC1, Histone deacetylase 1; MEF2A, Myocyte-specific enhancer factor 2A; RS6, 40S ribosomal protein S6; F262, 6-phosphofructo-2-kinase/fructose-2,6-bisphosphatase 2; ATRIP, ATM and Rad3-related-interacting protein; SHC1, Src homology 2 domain-containing-transforming protein C1; 4EBP1, Eukaryotic translation initiation factor 4E-binding protein 1; CCNY, Cyclin-Y; SRF, Serum response factor; HS90B, Heat shock protein HSP 90-beta; STMN1, Stathmin 1; WEE1, Wee1-like protein kinase; BRCA1, Breast cancer type 1 susceptibility protein; JUND, Transcription factor jun-D; EPB41, Erythrocyte membrane protein band 4.1; CDK7, Cyclin-dependent kinase 7; BAD, Bcl2-associated agonist of cell death; BCR, Breakpoint cluster region protein; G3BP1, Ras GTPase-activating protein-binding protein 1; ERBB2, Receptor tyrosine-protein kinase erbB-2; ACLY, ATP-citrate synthase; KSR1, Kinase suppressor of Ras 1; HS90A, Heat shock protein HSP 90-alpha; GAB1, Growth factor receptor bound protein 2-associated protein 1; HSF1, Heat shock factor protein 1; HNRPC, Heterogeneous nuclear ribonucleoproteins C1/C2; PP1R7, Protein phosphatase 1 regulatory subunit 7; TEBP, Prostaglandin E synthase 3; RBM8A, RNA-binding protein 8A; VIME, Vimentin; TBA1B, Tubulin alpha-1B chain.

F. Abbreviation for the common proteins identified in all four dataset as indicated in Figure 6C: PKD1(PRKD1), Protein kinase D1; SRC, Tyrosine-protein kinase Src; PAK2, p21 activated kinases 2; PEA15, Astrocytic phosphoprotein PEA-15; AKTS1, Proline-rich AKT1 substrate 1; GSK3A, Glycogen synthase kinase-3 alpha; CDK1, Cyclin-dependent kinase 1; EPB41, Erythrocyte membrane protein band 4.1; RS6, 40S ribosomal protein S6; HSF1, Heat shock factor protein 1; F262, 6-phosphofructo-2-kinase/fructose-2,6-bisphosphatase 2.

Figure S7

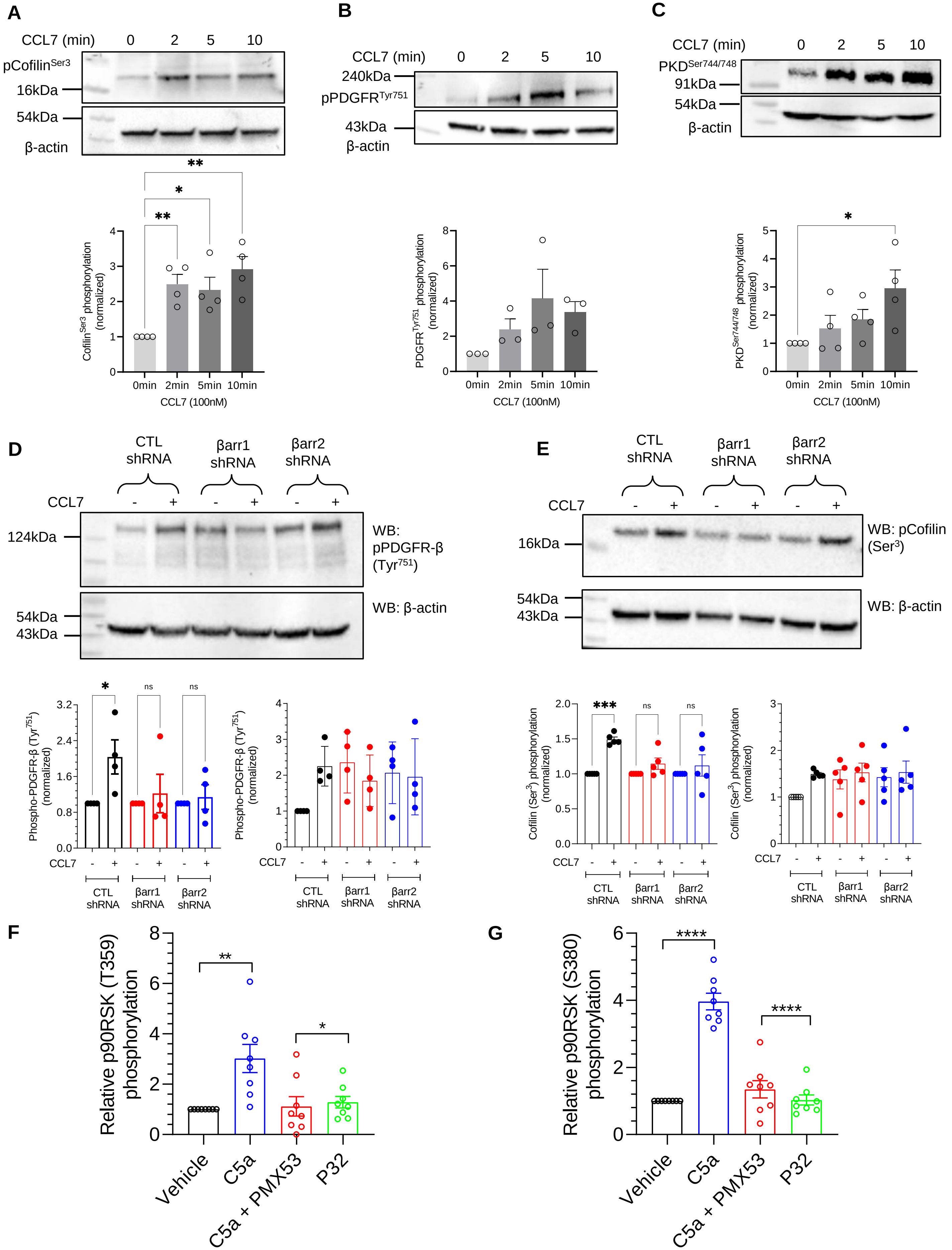

A
CCL7 (min)
0
2
5
10
pCofilinSer3
16kDa
54kDa
β-actin
**
*
**
Cofilin Ser3 phosphorylation (normalized)
0min
2min
5min
10min
CCL7 (100nM)
B
CCL7 (min)
240kDa
pPDGFRTyr751
43kDa
β-actin
PDGFRTyr751 phosphorylation (normalized)
C
PKDSer744/748
91kDa
54kDa
β-actin
PKDSer744/748 phosphorylation (normalized)
D
CTL shRNA
βarr1 shRNA
βarr2 shRNA
CCL7
124kDa
WB: pPDGFR-β (Tyr751)
54kDa
43kDa
WB: β-actin
ns
Phospho-PDGFR-β (Tyr751) (normalized)
E
16kDa
WB: pCofilin (Ser3)
***
Cofilin (Ser3) phosphorylation (normalized)
F
Relative p90RSK (T359) phosphorylation
Vehicle
C5a
C5a + PMX53
P32
G
****
Relative p90RSK (S380) phosphorylation

Figure S7, related to Figure 6 and 7. Validation of D6R and C5aR2 signaling.

A-C. Phosphorylation of cofilin, PDGFR-β and PKD1 upon CCL7-stimulation of D6R in HEK-293 cells was validated using Western blotting with corresponding antibodies. A representative blot from 3-5 experiments and densitometry-based quantification (mean ± SEM) is presented (*p<0.05, ***p<0.001; One-Way ANOVA).

D-E. shRNA-mediated depletion of βarr1 and βarr2 attenuate CCL7-induced (200nM) phosphorylation of PDGFR-β (Tyr751) and cofilin (Ser3). The upper panel shows a representative image from 4-5 independent experiments. The bar graphs on the left side of the lower panels show densitometry-based quantification, normalized with respect to the basal PDGFR-β and cofilin phosphorylation (i.e. without CCL7-stimulation) for each condition (i.e. CTL, βarr1 and βarr2 shRNA, and the bar graphs on the right side of the lower panel show the same data normalized differently i.e. with respect to the basal signal under control shRNA condition. Data are analyzed using One-Way ANOVA; *p<0.05, ***p<0.001).

F-G. The phosphorylation of p90RSK at Thr^{359} and Ser^{380} are primarily mediated through C5aR1 activation as pre-treatment with PMX53 (C5aR1 antagonist) blocks C5a-induced response. Moreover, P32 (C5aR2-specific agonist) stimulation of HMDMs also fails to induce significant phosphorylation on these sites. Phosphorylation of p90RSK at indicated sites was measured using in-cell ELISA assay with corresponding antibodies. Data from HMDMs derived from eight different donors were collected and normalized with respect to vehicle-treatment, and analyzed using Fisher's LSD test (*p<0.05, **p<0.01, ****p<0.0001), assuming each treatment group is independent of each other - only donors with a >1 response to C5a are included.
